# Reducing the worst case running times of a family of RNA and CFG problems, using Valiant's approach

**DOI:** 10.1186/1748-7188-6-20

**Published:** 2011-08-18

**Authors:** Shay Zakov, Dekel Tsur, Michal Ziv-Ukelson

**Affiliations:** 1 Department of Computer Science, Ben-Gurion University of the Negev, P.O.B. 653 Beer Sheva, 84105, Israel

## Abstract

**Background:**

RNA secondary structure prediction is a mainstream bioinformatic domain, and is key to computational analysis of functional RNA. In more than 30 years, much research has been devoted to defining different variants of RNA structure prediction problems, and to developing techniques for improving prediction quality. Nevertheless, most of the algorithms in this field follow a similar dynamic programming approach as that presented by Nussinov and Jacobson in the late 70's, which typically yields cubic worst case running time algorithms. Recently, some algorithmic approaches were applied to improve the complexity of these algorithms, motivated by new discoveries in the RNA domain and by the need to efficiently analyze the increasing amount of accumulated genome-wide data.

**Results:**

We study Valiant's classical algorithm for Context Free Grammar recognition in sub-cubic time, and extract features that are common to problems on which Valiant's approach can be applied. Based on this, we describe several problem templates, and formulate generic algorithms that use Valiant's technique and can be applied to all problems which abide by these templates, including many problems within the world of RNA Secondary Structures and Context Free Grammars.

**Conclusions:**

The algorithms presented in this paper improve the theoretical asymptotic worst case running time bounds for a large family of important problems. It is also possible that the suggested techniques could be applied to yield a practical speedup for these problems. For some of the problems (such as computing the RNA partition function and base-pair binding probabilities), the presented techniques are the only ones which are currently known for reducing the asymptotic running time bounds of the standard algorithms.

## 1 Background

RNA research is one of the classical domains in bioinformatics, receiving increasing attention in recent years due to discoveries regarding RNA's role in regulation of genes and as a catalyst in many cellular processes [1,2]. It is well-known that the function of an RNA molecule is heavily dependent on its structure [3]. However, due to the difficulty of *physically *determining RNA structure via wet-lab techniques, *computational prediction *of RNA structures serves as the basis of many approaches related to RNA functional analysis [4]. Most computational tools for RNA structural prediction focus on RNA *secondary structures *- a reduced structural representation of RNA molecules which describes a set of paired nucleotides, through hydrogen bonds, in an RNA sequence. RNA secondary structures can be relatively well predicted computationally in polynomial time (as opposed to three-dimensional structures). This computational feasibility, combined with the fact that RNA secondary structures still reveal important information about the functional behavior of RNA molecules, account for the high popularity of state-of-the-art tools for RNA secondary structure prediction [5].

Over the last decades, several variants of RNA secondary structure prediction problems were defined, to which polynomial algorithms have been designed. These variants include the basic *RNA folding *problem (predicting the secondary structure of a single RNA strand which is given as an input) [6-8], the *RNA-RNA Interaction *problem (predicting the structure of the complex formed by two or more interacting RNA molecules) [9], the *RNA Partition Function and Base Pair Binding Probabilities *problem of a single RNA strand [10] or an RNA duplex [11,12] (computing the pairing probability between each pair of nucleotides in the input), the *RNA Sequence to Structured-Sequence Alignment *problem (aligning an RNA sequence to sequences with known structures) [13,14], and the *RNA Simultaneous Alignment and Folding *problem (finding a secondary structure which is conserved by multiple homologous RNA sequences) [15]. Sakakibara et al. [16] noticed that the basic *RNA Folding *problem is in fact a special case of the *Weighted Context Free Grammar (WCFG) Parsing *problem (also known as *Stochastic *or *Probabilistic CFG Parsing*) [17]. Their approach was then followed by Dowell and Eddy [18], Do et al. [19], and others, who studied different aspects of the relationship between these two domains. The WCFG Parsing problem is a generalization of the simpler non-weighted *CFG Parsing *problem. Both WCFG and CFG Parsing problems can be solved by the Cocke-Kasami-Younger (CKY) dynamic programming algorithm [20-22], whose running time is cubic in the number of words in the input sentence (or in the number of nucleotides in the input RNA sequence).

The CFG literature describes two improvements which allow to obtain a sub-cubic time for the CKY algorithm. The first among these improvements was a technique suggested by Valiant [23], who showed that the CFG Parsing problem on a sentence with *n *words can be solved in a running time which matches the running time of a *Boolean Matrix Multiplication *of two *n *× *n *matrices. The current asymptotic running time bound for this variant of matrix multiplication was given by Coppersmith-Winograd [24], who showed an *O*(*n*^2.376^) time (theoretical) algorithm. In [25], Akutsu argued that the algorithm of Valiant can be modified to deal also with WCFG Parsing (this extension is described in more details in [26]), and consequentially with RNA Folding. The running time of the adapted algorithm is different from that of Valiant's algorithm, and matches the running time of a *Max-Plus Multiplication *of two *n *× *n *matrices. The current running time bound for this variant is On3log3 lognlog2n, given by Chan [27].

The second improvement to the CKY algorithm was introduced by Graham et al. [28], who applied the *Four Russians *technique [29] and obtained an $O\left(\frac{n^{3}}{\log\;n}\right)$ running time algorithm for the (non-weighted) CFG Parsing problem. To the best of our knowledge, no extension of this approach to the WCFG Parsing problem has been described. Recently, Frid and Gusfield [30] showed how to apply the *Four Russians *technique to the RNA folding problem (under the assumption of a discrete scoring scheme), obtaining the same running time of $O\left(\frac{n^{3}}{\log\;n}\right)$. This method was also extended to deal with the RNA simultaneous alignment and folding problem [31], yielding an $O\left(\frac{n^{6}}{\log\;n}\right)$ running time algorithm.

Several other techniques have been previously developed to accelerate the practical running times of different variants of CFG and RNA related algorithms. Nevertheless, these techniques either retain the same worst case running times of the standard algorithms [14,28,32-36], or apply heuristics which compromise the optimality of the obtained solutions [25,37,38]. For some of the problem variants, such as the *RNA Base Pair Binding Probabilities *problem (which is considered to be one of the variants that produces more reliable predictions in practice), no speedup to the standard algorithms has been previously described.

In his paper [23], Valiant suggested that his approach could be extended to additional related problems. However, in more than three decades which have passed since then, very few works have followed. The only extension of the technique which is known to the authors is Akutsu's extension to WCFG Parsing and RNA Folding [25,26]. We speculate that simplifying Valiant's algorithm would make it clearer and thus more accessible to be applied to a wider range of problems.

Indeed, in this work we present a simple description of Valiant's technique, and then further generalize it to cope with additional problem variants which do not follow the standard structure of CFG/WCFG Parsing (a preliminary version of this work was presented in [39]). More specifically, we define three template formulations, entitled *Vector Multiplication Templates (VMTs)*. These templates abstract the essential properties that characterize problems for which a Valiant-like algorithmic approach can yield algorithms of improved time complexity. Then, we describe generic algorithms for all problems sustaining these templates, which are based on Valiant's algorithm.

Table 1 lists some examples of VMT problems. The table compares between the running times of standard dynamic programming (DP) algorithms, and the VMT algorithms presented here. In the single string problems, *n *denotes the length of the input string. In the double-string problems [9,12,13], both input strings are assumed to be of the same length *n*. For the *RNA Simultaneous Alignment and Folding *problem, *m *denotes the number of input strings and *n *is the length of each string. *DB*(*n*) denotes the time complexity of a Dot Product or a Boolean Multiplication of two *n *× *n *matrices, for which the current best theoretical result is *O*(*n*^2.376^), due to Coppersmith and Winograd [24]. *MP*(*n*) denotes the time complexity of a Min-Plus or a Max-Plus Multiplication of two *n *× *n *matrices, for which the current best theoretical result is On3log3 lognlog2n, due to Chan [27]. For most of the problems, the algorithms presented here obtain lower running time bounds than the best algorithms previously known for these problems. It should be pointed out that the above mentioned matrix multiplication running times are the theoretical asymptotic times for sufficiently large matrices, yet they do not reflect the actual multiplication time for matrices of realistic sizes. Nevertheless, practical fast matrix multiplication can be obtained by using specialized hardware [40,41] (see Section 6).

**Table 1:** Time complexities of several VMT problems

	Problem	Standard DP running time	Implicit [explicit] VMT algorithm running time
Results previously published	CFG Recognition/Parsing	$\Theta (n^{3})$) [20-22]	$\Theta (DB(n))\left[\Theta \left(n^{2.38}\right)\right]$[23]
	
	WCFG Parsing	$\Theta (n^{3})$[17]	$\Theta (MP(n))\left[\tilde{O}\left(\frac{n^{3}}{\log^{2}\;n}\right)\right]$[25]
	
	RNA Single Strand Folding	$\Theta (n^{3})$[6,7]	$\Theta (MP(n))\left[\tilde{O}\left(\frac{n^{3}}{\log^{2}\;n}\right)\right]$[25]
	
	RNA Partition Function	$\Theta (n^{3})$[10]	$\Theta (MP(n))\left[\Theta \left(n^{2.38}\right)\right]$[25]

In this paper	WCFG Inside-Outside	$\Theta (n^{3})$[43]	$\Theta (DB(n))\left[\Theta \left(n^{2.38}\right)\right]$
	
	RNA Base Pair Binding Probabilities	$\Theta (n^{3})$[10]	$\Theta (DB(n))\left[\Theta \left(n^{2.38}\right)\right]$
	
	RNA Simultaneous Alignment and Folding	$\Theta \left({\left(n/2\right)}^{{}^{3m}}\right)$[15]	$\Theta (MP(n^{m}))[\tilde{O}(\frac{n^{3}m}{m\;\log^{2}\;n})]$
	
	RNA-RNA Interaction	$\Theta (n^{6})$[9]	$\Theta (MP(n^{2}))[\tilde{O}(\frac{n^{6}}{\log^{2}\;n})]$
	
	RNA-RNA Interaction Partition Function	$\Theta (n^{6})$[12]	$\Theta (DB(n))\left[\Theta \left(n^{4.75}\right)\right]$
	
	RNA Sequence to Structured-Sequence Alignment	$\Theta (n^{4})$[13]	$\Theta (nMP(n))[\tilde{O}(\frac{n^{4}}{\log^{2}\;n})]$

The notation $\tilde{O}$ corresponds to the standard big-*O *notation, hiding some polylogarithmic factors.

The formulation presented here has several advantages over the original formulation in [23]: First, it is considerably simpler, where the correctness of the algorithms follows immediately from their descriptions. Second, some requirements with respect to the nature of the problems that were stated in previous works, such as operation commutativity and distributivity requirements in [23], or the *semiring *domain requirement in [42], can be easily relaxed. Third, this formulation applies in a natural manner to algorithms for several classes of problems, some of which we show here. Additional problem variants which do not follow the exact templates presented here, such as the formulation in [12] for the RNA-RNA Interaction Partition Function problem, or the formulation in [13] for the RNA Sequence to Structured-Sequence Alignment problem, can be solved by introducing simple modifications to the algorithms we present. Interestingly, it turns out that almost every variant of RNA secondary structure prediction problem, as well as additional problems from the domain of CFGs, sustain the VMT requirements. Therefore, Valiant's technique can be applied to reduce the worst case running times of a large family of important problems. In general, as explained later in this paper, VMT problems are characterized in that their computation requires the execution of many vector multiplication operations, with respect to different multiplication variants (*Dot Product*, *Boolean Multiplication*, and *Min/Max Plus Multiplication*). Naively, the time complexity of each vector multiplication is linear in the length of the multiplied vectors. Nevertheless, it is possible to organize these vector multiplications as parts of square matrix multiplications, and to apply fast matrix multiplication algorithms in order to obtain a sub-linear (amortized) running time for each vector multiplication. As we show, a main challenge in algorithms for VMT problems is to describe how to bundle subsets of vector multiplications operations in order to compute them via the application of fast matrix multiplication algorithms. As the elements of these vectors are computed along the run of the algorithm, another aspect which requires attention is the decision of the order in which these matrix multiplications take place.

### Road Map

In Section 2 the basic notations are given. In Section 3 we describe the *Inside Vector Multiplication Template *- a template which extracts features for problems to which Valiant's algorithm can be applied. This section also includes the description of an exemplary problem (Section 3.1), and a generalized and simplified exhibition of Valiant's algorithm and its running time analysis (Section 3.3). In Sections 4 and 5 we define two additional problem templates: the *Outside Vector Multiplication Template*, and the *Multiple String Vector Multiplication Template*, and describe modifications to the algorithm of Valiant which allow to solve problems that sustain these templates. Section 6 concludes the paper, summarizing the main results and discussing some of its implications. Two additional exemplary problems (an Outside and a Multiple String VMT problems) are presented in the Appendix.

## 2 Preliminaries

As intervals of integers, matrices, and strings will be extensively used throughout this work, we first define some related notation.

### 2.1 Interval notations

**Figure 1:**
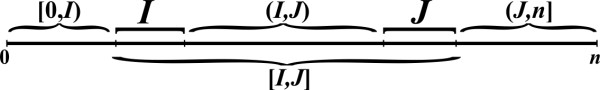
**Some interval examples**.

For two integers *a*, *b*, denote by [*a*, *b*] the interval which contains all integers *q *such that *a *≤ *q *≤ *b*. For two intervals *I *= [*i*_1_, *i*_2_] and *J *= [*j*_1_, *j*_2_], define the following intervals: [*I*, *J*] = {*q *: *i*_1 _≤ *q *≤ *j*_2_}, (*I*, *J*) = {*q *: *i*_2 _*< q < j*_1_}, [*I*, *J*) = {*q *: *i*_1 _≤ *q < j*_1_}, and (*I*, *J*] = {*q *: *i*_2 _*< q *≤ *j*_2_} (Figure 1). When an integer *r *replaces one of the intervals *I *or *J *in the notation above, it is regarded as the interval [*r*, *r*]. For example, [0, *I*) = {*q *: 0 ≤ *q < i*_1_}, and (*i*, *j*) = {*q *: *i < q < j*}. For two intervals *I *= [*i*_1_, *i*_2_] and *J *= [*j*_1_, *j*_2_] such that *j*_1 _= *i*_2 _+ 1, define *IJ *to be the concatenation of *I *and *J*, i.e. the interval [*i*_1_, *j*_2_].

### 2.2 Matrix notations

Let *X *be an *n*_1 _× *n*_2 _matrix, with rows indexed with 0, 1, ..., *n*_1 _- 1 and columns indexed with 0, 1, ..., *n*_2 _- 1. Denote by *X*_*i*, *j *_the element in the *i*th row and *j*th column of *X*. For two intervals *I *⊆ [0, *n*_1_) and *J *⊆ [0, *n*_2_), let *X*_*I*, *J *_denote the sub-matrix of *X *obtained by projecting it onto the subset of rows *I *and subset of columns *J*. Denote by *X*_*i*, *J *_the sub-matrix *X*_[*i*,*i*],*J*_, and by *X*_*I*, *j *_the sub-matrix *X*_*I*,[*j*,*j*]_. Let  D be a domain of elements, and ⊗ and ⊕ be two binary operations on  D. We assume that (1) ⊕ is associative (i.e. for three elements *a*, *b*, *c *in the domain, (*a *⊕ *b*) ⊕ *c *= *a *⊕ (*b *⊕ *c*)), and (2) there exists a *zero *element *ϕ *in  D, such that for every element a∈D*a *⊕ *ϕ *= *ϕ *⊕ *a *= *a *and *a *⊗ *ϕ *= *ϕ *⊗ *a *= *ϕ*.

Let *X *and *Y *be a pair of matrices of sizes *n*_1 _× *n*_2 _and *n*_2 _× *n*_3_, respectively, whose elements are taken from  D. Define the result of the *matrix multiplication X *⊗ *Y *to be the matrix *Z *of size *n*_1 _× *n*_3_, where each entry *Z*_*i*, *j *_is given by

$$Z_{i,j}={⊕}_{q\in \left[0,n_{2}\right)}\left(X_{i,q}⊗Y_{q,j}\right)=\left(X_{i,0}⊗Y_{0,j}\right)⊕\left(X_{i,1}⊗Y_{1,j}\right)⊕\ldots ⊕\left(X_{i,n_{2}-1}⊗Y_{n_{2}-1,j}\right).$$

In the special case where *n*_2 _= 0, define the result of the multiplication *Z *to be an *n*_1 _× *n*_3 _matrix in which all elements are *ϕ*. In the special case where *n*_1 _= *n*_3 _= 1, the matrix multiplication *X *⊗ *Y *is also called a *vector multiplication *(where the resulting matrix *Z *contains a single element).

Let *X *and *Y *be two matrices. When *X *and *Y *are of the same size, define the result of the *matrix addition X *⊕ *Y *to be the matrix *Z *of the same size as *X *and *Y*, where *Z*_*i*, *j *_= *X*_*i*, *j *_⊕ *Y*_*i*, *j*_. When *X *and *Y *have the same number of columns, denote by $\left[\begin{array}{c} X\\ Y \end{array}\right]$ the matrix obtained by concatenating *Y *below *X*. When *X *and *Y *have the same number of rows, denote by [*XY*] the matrix obtained by concatenating *Y *to the right of *X*. The following properties can be easily deduced from the definition of matrix multiplication and the associativity of the ⊕ operation (in each property the participating matrices are assumed to be of the appropriate sizes).

(1) $$\left[\begin{array}{c} X^{1}\\ X^{2} \end{array}\right]⊗Y\;=\;\left[\begin{array}{c} X^{1}⊗Y\\ X^{2}⊗Y \end{array}\right]$$

(2) $$X⊗\left[Y^{1}Y^{2}\right]\;=\;\left[\left(X⊗Y^{1}\right)\left(X⊗Y^{2}\right)\right]$$

(3) $$\left(X^{1}⊗Y^{1}\right)⊕\left(X^{2}⊗Y^{2}\right)\;=\;\left[X^{1}X^{2}\right]⊗\left[\begin{array}{c} Y^{1}\\ Y^{2} \end{array}\right]$$

Under the assumption that the operations ⊗ and ⊕ between two domain elements consume Θ(1) computation time, a straightforward implementation of a matrix multiplication between two *n *× *n *matrices can be computed in Θ(*n*^3^) time. Nevertheless, for some variants of multiplications, sub-cubic algorithms for square matrix multiplications are known. Here, we consider three such variants, which will be referred to as *standard multiplications *in the rest of this paper:

• *Dot Product*: The matrices hold numerical elements, ⊗ stands for number multiplication (·) and ⊕ stands for number addition (+). The *zero *element is 0. The running time of the currently fastest algorithm for this variant is *O*(*n*^2.376^) [24].

• *Min-Plus/Max-Plus Multiplication*: The matrices hold numerical elements,⊗ stands for number addition and ⊕ stands for min or max (where *a *min *b *is the minimum between *a *and *b*, and similarly for max). The *zero *element is ∞ for the Min-Plus variant and -∞ for the Max-Plus variant. The running time of the currently fastest algorithm for these variants is On3log3 lognlog2n[27].

• *Boolean Multiplication*: The matrices hold boolean elements, ⊗ stands for *boolean AND *(⋀) and ⊕ stands for *boolean OR*(⋁). The *zero *element is the *false *value. Boolean Multiplication is computable with the same complexity as the Dot Product, having the running time of *O*(*n*^2.376^) [24].

### 2.3 String notations

Let *s *= *s*_0_*s*_1 _... *s*_*n *- 1 _be a string of length *n *over some alphabet. A *position q *in *s *refers to a point between the characters *s*_*q *- 1 _and *s_q _*(a position may be visualized as a vertical line which separates between these two characters). Position 0 is regarded as the point just before *s*_0_, and position *n *as the point just after *s*_*n *- 1_. Denote by ||*s*|| = *n *+ 1 the number of different positions in *s*. Denote by *s*_*i*, *j *_the substring of *s *between positions *i *and *j*, i.e. the string *s*_*i *_*s*_*i*+1 _... *s*_*j *- 1_. In a case where *i *= *j*, *s*_*i*, *j *_corresponds to an empty string, and for *i > j*, *s*_*i*, *j *_does not correspond to a valid string.

**Figure 2:**
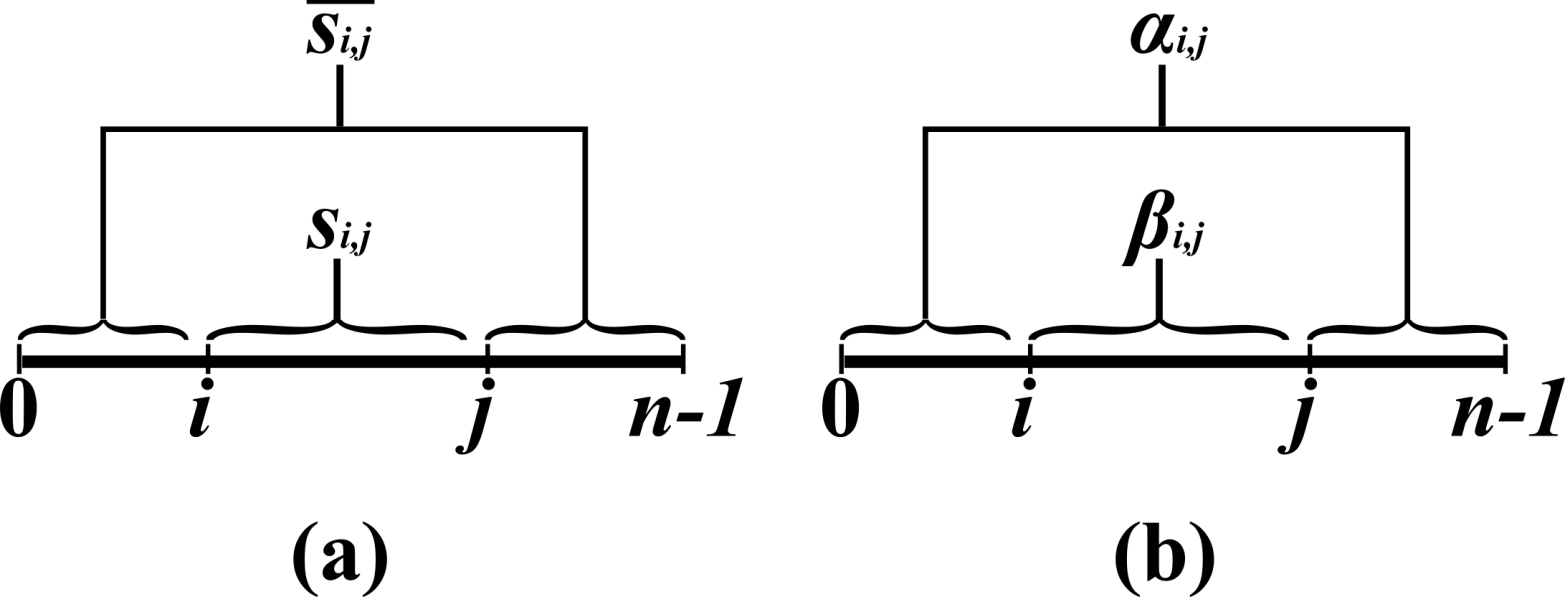
**Inside and outside sub-instances, and their corresponding properties**.

An *inside property β*_*i*,*j *_is a property which depends only on the substring *s*_*i*, *j *_(Figure 2). In the context of RNA, an input string usually represents a sequence of nucleotides, where in the context of CFGs, it usually represents a sequence of words. Examples of inside properties in the world of RNA problems are the maximum number of base-pairs in a secondary structure of *s*_*i*, *j *_[6], the minimum free energy of a secondary structure of *s*_*i*, *j *_[7], the sum of weights of all secondary structures of *s*_*i*, *j *_[10], etc. In CFGs, inside properties can be boolean values which state whether the sub-sentence can be derived from some non-terminal symbol of the grammar, or numeric values corresponding to the weight of (all or best) such derivations [17,20-22].

An *outside property α*_*i*,*j *_is a property of the residual string obtained by removing *s*_*i*, *j *_from *s *(i.e. the pair of strings *s*_0,*i *_and *s*_*j*,*n*_, see Figure 2). Such a residual string is denoted by $\bar{s_{i,j}}$. Outside property computations occur in algorithms for the RNA Base Pair Binding Probabilities problem [10], and in the Inside-Outside algorithm for learning derivation rule weights for WCFGs [43].

In the rest of this paper, given an instance string *s*, substrings of the form *s*_*i*, *j *_and residual strings of the form $\bar{s_{i,j}}$ will be considered as *sub-instances *of *s*. Characters and positions in such sub-instances are indexed according to the same indexing as of the original string *s*. That is, the characters in sub-instances of the form *s*_*i*, *j *_are indexed from *i *to *j *- 1, and in sub-instances of the form $\bar{s_{i,j}}$ the first *i *characters are indexed between 0 and *i *- 1, and the remaining characters are indexed between *j *and *n *- 1. The notation *β *will be used to denote the set of all values of the form *β*_*i*,*j *_with respect to substrings *s*_*i*, *j *_of some given string *s*. It is convenient to visualize *β *as an ||*s*|| × ||*s*|| matrix, where the (*i*, *j*)-th entry in the matrix contains the value *β*_*i*,*j*_. Only entries in the upper triangle of the matrix *β *correspond to valid substrings of *s*. For convenience, we define that values of the form *β*_*i*, *j*_, when *j < i*, equal to *ϕ *(with respect to the corresponding domain of values). Notations such as *β*_*I*, *J*_, *β*_*i*, *J*_, and *β*_*I*, *j *_are used in order to denote the corresponding sub-matrices of *β*, as defined above. Similar notations are used for a set *α *of outside properties.

## 3 The Inside Vector Multiplication Template

In this section we describe a template that defines a class of problems, called the *Inside Vector Multiplication Template *(*Inside VMT*). We start by giving a simple motivating example in Section 3.1. Then, the class of Inside VMT problems is formally defined in Section 3.2, and in Section 3.3 an efficient generic algorithm for all Inside VMT problems is presented.

### 3.1 Example: RNA Base-Pairing Maximization

The *RNA Base-Pairing Maximization *problem [6] is a simple variant of the *RNA Folding *problem, and it exhibits the main characteristics of Inside VMT problems. In this problem, an input string *s *= *s*_0_*s*_1 _⋯ *s*_*n *- 1 _represents a string of *bases *(or *nucleotides*) over the alphabet *A*, *C*, *G*, *U*. Besides strong (covalent) chemical bonds which occur between each pair of consecutive bases in the string, bases at distant positions tend to form additional weaker (hydrogen) bonds, where a base of type *A *can pair with a base of type *U*, a base of type *C *can pair with a base of type *G*, and in addition a base of type *G *can pair with a base of type *U*. Two bases which can pair to each other in such a (weak) bond are called *complementary bases*, and a bond between two such bases is called a *base-pair*. The notation *a *• *b *is used to denote that the bases at indices *a *and *b *in *s *are paired to each other.

**Figure 3:**
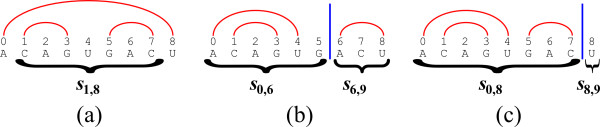
**An RNA string *s *= *s*_0,9 _= *ACAGUGACU*, and three corresponding foldings**. (a) A folding of type *I*, obtained by adding the base-pair *i *• (*j *- 1) = 0 • 8 to a folding for *s*_*i*+1, *j*-1 _= *s*_1,8_. (b) A folding of type *II*, in which the last index 8 is paired to index 6. The folding is thus obtained by combining two independent foldings: one for the prefix *s*_0,6_, and one for the suffix *s*_6,9_. (c) A folding of type *II*, in which the last index 8 is unpaired. The folding is thus obtained by combining a folding for the prefix *s*_0,8_, and an empty folding for the suffix *s*_8,9_.

A *folding *(or a *secondary structure*) of *s *is a set *F *of base-pairs of the form *a *• *b*, where 0 ≤ *a < b < n*, which sustains that there are no two distinct base pairs *a *• *b *and *c *• *d *in *F *such that *a *≤ *c *≤ *b *≤ *d *(i.e. the paring is nested, see Figure 3). Denote by |*F*| the number of complementary base-pairs in *F*. The goal of the RNA base-paring maximization problem is to compute the maximum number of complementary base-pairs in a folding of an input RNA string *s*. We call such a number the *solution *for *s*, and denote by *β*_*i*, *j *_the solution for the substring *s*_*i*,*j*_. For substrings of the form *s*_*i*, *i *_and *s*_*i*, *i*+1 _(i.e. empty strings or strings of length 1), the only possible folding is the empty folding, and thus *β*_*i*, *i *_= *β*_*i*, *i*+1 _= 0. We next explain how to recursively compute *β*_*i*, *j *_when *j > i *+ 1.

In order to compute values of the form *β*_*i*, *j*_, we distinguish between two types of foldings for a substring *s*_*i*, *j*_: foldings of type *I *are those which contain the base-pair *i *• (*j *- 1), and foldings of type *II *are those which do not contain *i *• (*j *- 1).

Consider a folding *F *of type *I*. Since *i *• (*j *- 1) ∈ *F*, the folding *F *is obtained by adding the base-pair *i *• (*j - *1) to some folding *F' *for the substring *s*_*i*+1, *j *- 1 _(Figure 3a). The number of complementary base-pairs in *F *is thus |*F'*| + 1 if the bases *s_i _*and *s*_*j *- 1 _are complementary, and otherwise it is |*F'*|. Clearly, the number of complementary base-pairs in *F *is maximized when choosing *F' *such that |*F'*| = *β*_*i*+1, *j *- 1_. Now, Consider a folding *F *of type *II*. In this case, there must exist some position *q *∈ (*i*, *j*), such that no base-pair *a *• *b *in *F *sustains that *a < q *≤ *b*. This observation is true, since if *j *- 1 is paired to some index *p *(where *i < p < j *- 1), then *q *= *p *sustains the requirement (Figure 3b), and otherwise *q *= *j *- 1 sustains the requirement (Figure 3c). Therefore, *q *splits *F *into two independent foldings: a folding *F' *for the prefix *s*_*i*, *q*_, and a folding *F" *for the suffix *s*_*q*, *j*_, where |*F*| = |*F'*| + |*F"*|. For a specific split position *q*, the maximum number of complementary base-pairs in a folding of type *II *for *s*_*i*, *j *_is then given by *β*_*i*, *q *_+ *β*_*q*, *j*_, and taking the maximum over all possible positions *q *∈ (*i*, *j*) guarantees that the best solution of this form is found.

Thus, *β*_*i*, *j *_can be recursively computed according to the following formula:

$$\beta_{i,j}=\max\left\{\begin{array}{cc} \left(I\right) & \beta_{i+1,j-1}+\delta_{i,j-1},\\ \left(II\right) & \underset{q\in \left(i,j\right)}{\max}\left\{\beta_{i,q}+\beta_{q,j}\right\} \end{array}\right\},$$

where *δ*_*i*, *j - *1 _= 1 if *s_i _*and *s*_*j *- 1 _are complementary bases, and otherwise *δ*_*i*,*j *- 1 _= 0.

#### 3.1.1 The classical algorithm

**Figure 4:**
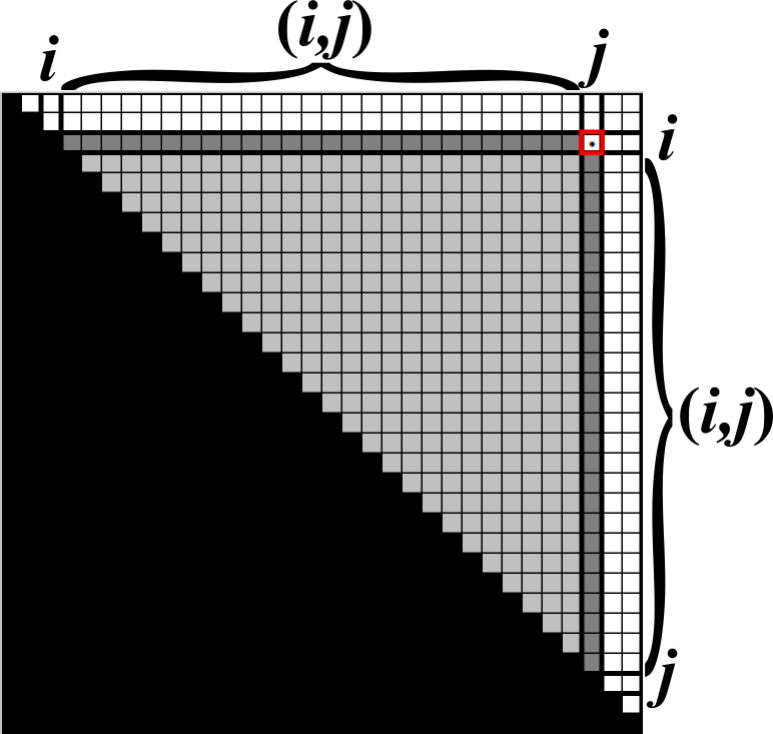
**The table *B *maintained by the DP algorithm**. In order to compute *B*_*i*, *j*_, the algorithm needs to examine only values in entries of *B *that correspond to strict substrings of *s*_*i*, *j *_(depicted as light and dark grayed entries).

The recursive computation above can be efficiently implemented using dynamic programming (DP). For an input string *s *of length *n*, the DP algorithm maintains the upper triangle of an ||*s*|| × ||*s*|| matrix *B*, where each entry *B*_*i*, *j *_in *B *corresponds to a solution *β*_*i*, *j*_. The entries in *B *are filled, starting from short base-case entries of the form *B*_*i*, *i *_and *B*_*i*,*i*+1_, and continuing by computing entries corresponding to substrings with increasing lengths. In order to compute a value *β*_*i*, *j *_according to the recurrence formula, the algorithm needs to examine only values of the form *β*_*i'*,*j' *_such that *s*_*i'*,*j' *_is a strict substring of *s*_*i*, *j *_(Figure 4). Due to the bottom-up computation, these values are already computed and stored in *B*, and thus each such value can be obtained in Θ(1) time.

Upon computing a value *β*_*i*, *j*_, the algorithm needs to compute term (*II*) of the recurrence. This computation is of the form of a *vector multiplication operation *⊕_*q*∈(*i*,*j*) _(*β_i, q _*⊗ *β*_*q*, *j*_), where the multiplication variant is the *Max Plus *multiplication. Since all relevant values in *B *are computed, this computation can be implemented by computing *B*_*i*, (*i*,*j*) _⊗ *B*_(*i*,*j*),*j *_(the multiplication of the two darkened vectors in Figure 4), which takes Θ(*j *- *i*) running time. After computing term (*II*), the algorithm needs to perform additional operations for computing *β*_*i*, *j *_which take Θ(1) running time (computing term (*I*), and taking the maximum between the results of the two terms). It can easily be shown that, on average, the running time for computing each value *β*_*i*, *j *_is Θ(*n*), and thus the overall running time for computing all Θ(*n*^2^) values *β*_*i*, *j *_is Θ(*n*^3^). Upon termination, the computed matrix *B *equals to the matrix *β*, and the required result *β*_0,*n *_is found in the entry *B*_0,*n*_.

### 3.2 Inside VMT definition

In this section we characterize the class of *Inside VMT *problems. The *RNA Base-Paring Maximization *problem, which was described in the previous section, exhibits a simple special case of an Inside VMT problem, in which the goal is to compute a single inside property for a given input string. Note that this requires the computation of such inside properties for all substrings of the input, due to the recursive nature of the computation. In other Inside VMT problems the case is similar, hence we will assume that the goal of Inside VMT problems is to compute inside properties for *all *substrings of the input string. In the more general case, an Inside VMT problem defines several inside properties, and all of these properties are computed for each substring of the input in a mutually recursive manner. Examples of such problems are the *RNA Partition Function *problem [10] (which is described in Appendix A), the *RNA Energy Minimization *problem [7] (which computes several folding scores for each substring of the input, corresponding to restricted types of foldings), and the *CFG Parsing *problem [20-22] (which computes, for every non-terminal symbol in the grammar and every sub-sentence of the input, a boolean value that indicates whether the sub-sentence can be derived in the grammar when starting the derivation from the non-terminal symbol).

A common characteristic of all Inside VMT problems is that the computation of at least one type of an inside property requires a result of a vector multiplication operation, which is of similar structure to the multiplication described in the previous section for the RNA Base-Paring Maximization problem. In many occasions, it is also required to output a *solution *that corresponds to the computed property, e.g. a minimum energy secondary structure in the case of the RNA folding problem, or a maximum weight parse-tree in the case of the WCFG Parsing problem. These solutions can usually be obtained by applying a traceback procedure over the computed dynamic programming tables. As the running times of these traceback procedures are typically negligible with respect to the time needed for filling the values in the tables, we disregard this phase of the computation in the rest of the paper.

The following definition describes the family of *Inside VMT problems*, which share common combinatorial characteristics and may be solved by a generic algorithm which is presented in Section 3.3.

**Definition 1 ***A problem is considered an *Inside VMT problem *if it fulfills the following requirements*.

*1. Instances of the problem are strings, and the goal of the problem is to compute for every substring s*_*i*, *j*_* of an input string s, a series of inside properties *$\beta_{i,j}^{1},\;\beta_{i,j}^{2},\;\ldots ,\;\beta_{i,j}^{K}$.

*2. Let s*_*i*, *j*_* be a substring of some input string s. Let *1 ≤ *k *≤ *K, and let *$\mu_{i,j}^{k}$*be a result of a vector multiplication of the form *$\mu_{i,j}^{k}={⊕}_{q\in \left(i,j\right)}\left(\beta_{i,q}^{k^{\prime }}⊗\beta_{q,j}^{k^{\prime \prime }}\right)$*, for some *1 ≤ *k'*, *k" *≤ *K. Assume that the following values are available: *$\mu_{i,j}^{k}$*, all values *$\beta_{i^{\prime },j^{\prime }}^{k^{\prime }}$*for *1 ≤ *k' *≤ *K and s*_*i'*,*j'*_* a strict substring of s*_*i*, *j*_, and all values $\beta_{i,j}^{k^{\prime }}$*for *1 ≤ *k' < k. Then, *$\beta_{i,j}^{k}$*can be computed in o*(||*s*||) *running time*.

*3. In the multiplication variant that is used for computing *$\mu_{i,j}^{k}$*, the *⊕ *operation is associative, and the domain of elements contains a zero element. In addition, there is a matrix multiplication algorithm for this multiplication variant, whose running time M*(*n*) *over two n *× *n matrices satisfies M*(*n*) = *o*(*n*^3^).

**Figure 5:**
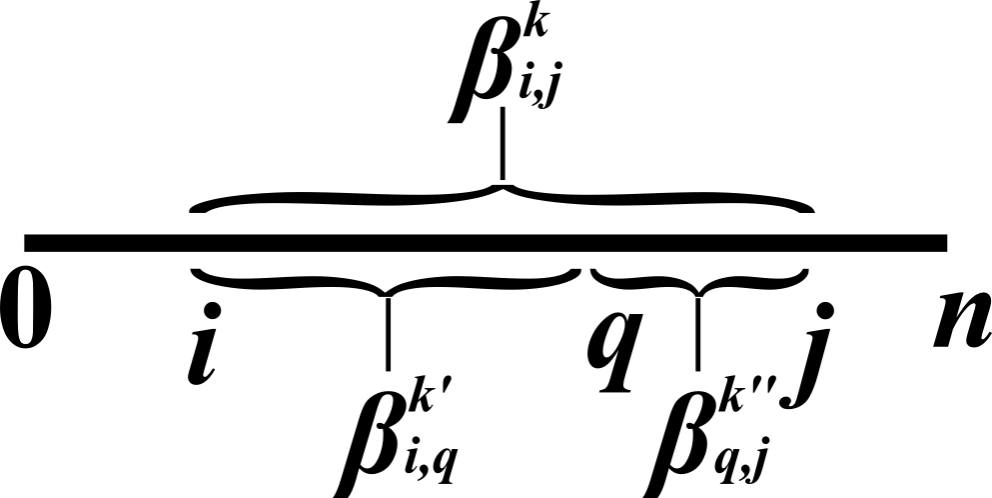
**The examination of a split position *q *in the computation of an inside property $\beta_{i,j}^{k}$**.

Intuitively, $\mu_{i,j}^{k}$ reflects an expression which examines all possible splits of *s*_*i*, *j *_into a prefix substring *s*_*i*, *q *_and a suffix substring *s*_*q*, *j *_(Figure 5). Each split corresponds to a term that should be considered when computing the property $\beta_{i,j}^{k}$, where this term is defined to be the application of the ⊗ operator between the property $\beta_{i,q}^{k^{\prime }}$ of the prefix *s*_*i*, *q*_, and the property $\beta_{q,j}^{k^{\prime \prime }}$ of the suffix *s*_*q*, *j *_(where ⊗ usually stands for +, ·, or ⋀). The combined value $\mu_{i,j}^{k}$ for all possible splits is then defined by applying the ⊕ operation (usually min/max, +, or ⋁) over these terms, in a sequential manner. The template allows examining $\mu_{i,j}^{k}$, as well as additional values of the form $\beta_{i^{\prime },j^{\prime }}^{k^{\prime }}$, for strict substrings *s*_*i'*,*j' *_of *s*_*i*, *j *_and 1 ≤ *k' *<*K*, and values of the form $\beta_{i,j}^{k^{\prime }}$ for 1 ≤ *k' < k*, in order to compute $\beta_{i,j}^{k}$. In typical VMT problems (such as the RNA Base-Paring Maximization problem, and excluding problems which are described in Section 5), the algorithm needs to perform Θ(1) operations for computing $\beta_{i,j}^{k}$, assuming that $\mu_{i,j}^{k}$ and all other required values are pre-computed. Nevertheless, the requirement stated in the template is less strict, and it is only assumed that this computation can be executed in a sub-linear time with respect to ||*s*||.

### 3.3 The Inside VMT algorithm

We next describe a generic algorithm, based on Valiant's algorithm [23], for solving problems sustaining the Inside VMT requirements. For simplicity, it is assumed that a single property *β*_*i*, *j *_needs to be computed for each substring *s*_*i*, *j *_of the input string *s*. We later explain how to extend the presented algorithm to the more general cases of computing *K *inside properties for each substring.

**Figure 6:**
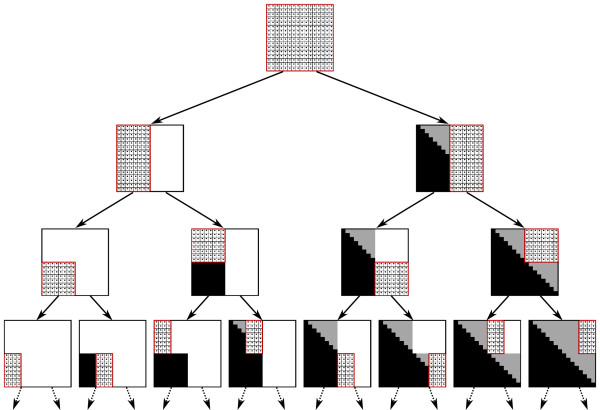
**The recursion tree**. Each node in the tree shows the state of the matrix *B *when the respective call to *Compute-Inside-Sub-Matrix *starts. The dotted cells are those that are computed during the call. Black and gray cells are cells whose values were already computed (black cells correspond to empty substrings). The algorithm starts by calling the recursive procedure over the complete matrix. Each visited sub-matrix is decomposed into two halves, which are computed recursively. The recursion visits the sub-matrices according to a pre-order scan on the tree depicted in the figure. Once the first among a pair of sibling recursive calls was concluded, the algorithm uses the new computed portion of data as an input to a fast matrix multiplication subroutine, which facilitate the computation of the second sibling.

The new algorithm also maintains a matrix *B *as defined in Section 3.1. It is a divide-and-conquer recursive algorithm, where at each recursive call the algorithm computes the values in a sub-matrix *B*_*I*, *J *_of *B *(Figure 6). The actual computation of values of the form *β*_*i*, *j *_is conducted at the base-cases of the recurrence, where the corresponding sub-matrix contains a single entry *B*_*i*, *j*_. The main idea is that upon reaching this stage the term *μ*_*i*, *j *_was already computed, and thus *β*_*i*, *j *_can be computed efficiently, as implied by item 2 of Definition 1. The accelerated computation of values of the form *μ*_*i*, *j *_is obtained by the application of fast matrix multiplication subroutines between sibling recursive calls of the algorithm. We now turn to describe this process in more details.

**Figure 7:**
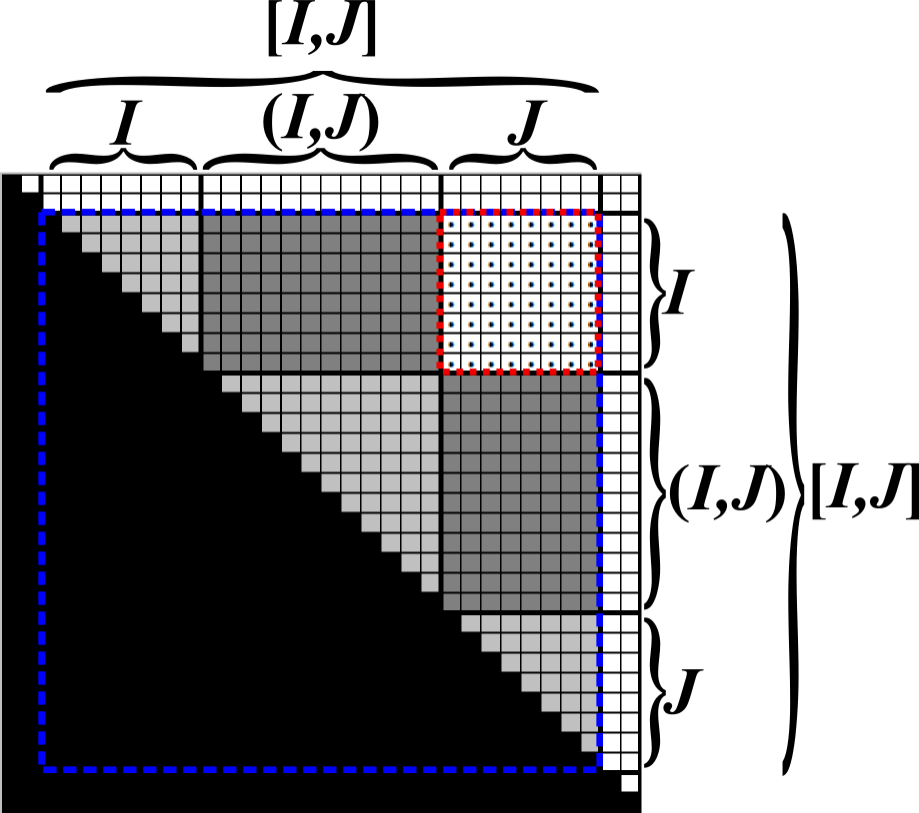
**The pre-condition for computing *B*_*I*, *J *_with the Inside VMT algorithm**. All values in *B*_[*I*,*J*], [*I*,*J*]_, excluding *B*_*I*, *J*_, are computed (light and dark grayed entries), and *B*_*I*, *J *_= *β*_*I*,(*I*,*J*) _⊗ *β *_(*I*,*J*),*J *_= *B*_*I*,(*I*,*J*) _⊗ *B_(I, J), J _*(the entries of *B*_*I*,(*I*,*J*) _and *B*_(*I*,*J*),*J *_are dark grayed).

At each stage of the run, each entry *B*_*i*, *j *_either contains the value *β*_*i*,*j*_, or some intermediate result in the computation of *μ*_*i*, *j*_. Note that only the upper triangle of *B *corresponds to valid substrings of the input. Nevertheless, our formulation handles all entries uniformly, implicitly ignoring values in entries *B*_*i*, *j *_when *j < i*. The following pre-condition is maintained at the beginning of the recursive call for computing *B*_*I*, *J *_(Figure 7):

1. Each entry *B*_*i*, *j *_in *B*_[*I*,*J*], [*I*,*J*] _contains the value *β*_*i*, *j*_, except for entries in *B*_*I*, *J*_.

2. Each entry *B*_*i*, *j *_in *B*_*I*, *J *_contains the value ⊕_*q*∈(*I*,*J*) _(*β*_*i*, *q *_⊗ *β*_*q*, *j*_). In other words, *B*_*I*, *J *_= *β*_*I*,(*I*,*J*) _⊗ *β*_(*I*,*J*),*J*_.

Let *n *denote the length of *s*. Upon initialization, *I *= *J *= [0, *n*], and all values in *B *are set to *ϕ*. At this stage (*I*, *J*) is an empty interval, and so the pre-condition with respect to the complete matrix *B *is met. Now, consider a call to the algorithm with some pair of intervals *I*, *J*. If *I *= [*i*, *i*] and *J *= [*j*, *j*], then from the pre-condition, all values *β*_*i'*, *j' *_which are required for the computation *β*_*i*, *j *_of are computed and stored in *B*, and *B*_*i*, *j *_= *μ*_*i*, *j *_(Figure 4). Thus, according to the Inside VMT requirements, *β*_*i*, *j *_can be evaluated in *o*(||*s*||) running time, and be stored in *B*_*i*, *j*_.

**Figure 8:**
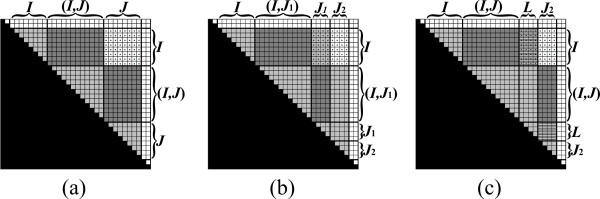
**An exemplification of the vertical partition of *B*_*I*, *J *_(the entries of *B*_*I*, *J *_are dotted)**. (a) The pre-condition requires that all values in *B*_[*I*,*J*], [*I*,*J*]_, excluding *B*_*I*, *J*_, are computed, and *B_I, J _*= *β*_*I*,(*I*,*J*) _⊗ *β *_(*I*,*J*),*J *_(see Figure 7). (b) *B_I, J _*is partitioned vertically to $B_{I,J_{1}}$ and $B_{I,J_{2}}$, where $B_{I,J_{1}}$ is computed recursively. (c) The pre-condition for computing $B_{I,J_{2}}$ is established, by updating $B_{I,J_{2}}$ to be $B_{I,J_{2}}⊕\left(B_{I,L}⊗B_{L,J_{2}}\right)$ (in this example *L *= *J*_1_, since *I *ends before *J*_1 _starts). Then, $B_{I,J_{2}}$ is computed recursively (not shown).

Else, either |*I*| *>*1 or |*J*| *>*1 (or both), and the algorithm partitions *B*_*I*, *J *_into two sub-matrices of approximately equal sizes, and computes each part recursively. This partition is described next. In the case where |*I*| ≤ |*J*|, *B*_*I*, *J *_is partitioned vertically (Figure 8). Let *J*_1 _and *J*_2 _be two column intervals such that *J *= *J*_1_*J*_2 _and |*J*_1_| = ⌊|*J*|/2⌋ (Figure 8b). Since *J *and *J*_1 _start at the same index, (*I*, *J*) = (*I*, *J*_1_). Thus, from the pre-condition and Equation 2, $B_{I,J_{1}}=\beta_{I,\left(I,J_{1}\right)}⊗\beta_{\left(I,J_{1}\right),J_{1}}$. Therefore, the pre-condition with respect to the sub-matrix $B_{I,J_{1}}$ is met, and the algorithm computes this sub-matrix recursively. After $B_{I,J_{1}}$ is computed, the first part of the pre-condition with respect to $B_{I,J_{2}}$ is met, i.e. all necessary values for computing values in $B_{I,J_{2}}$, except for those in $B_{I,J_{2}}$ itself, are computed and stored in *B*. In addition, at this stage $B_{I,J_{2}}=\beta_{I,\left(I,J\right)}⊗\beta_{\left(I,J\right),J_{2}}$. Let *L *be the interval such that (*I*, *J*_2_) = (*I*, *J*)*L*. *L *is contained in *J*_1_, where it can be verified that either *L *= *J*_1 _(if the last index in *I *is smaller than the first index in *J*, as in the example of Figure 8c), or *L *is an empty interval (in all other cases which occur along the recurrence). To meet the full pre-condition requirements with respect to *I *and *J*_2_, $B_{I,J_{2}}$ is updated using Equation 3 to be $B_{I,J_{2}}⊕\left(B_{I,L}⊗B_{L,J_{2}}\right)=\left(\beta_{I,\left(I,J\right)}⊗\beta_{\left(I,J\right),J_{2}}\right)⊕\left(\beta_{I,L}⊗\beta_{L,J_{2}}\right)=\beta_{I,\left(I,J_{2}\right)}⊗\beta_{\left(I,J_{2}\right),J_{2}}$.

**Figure 9:**
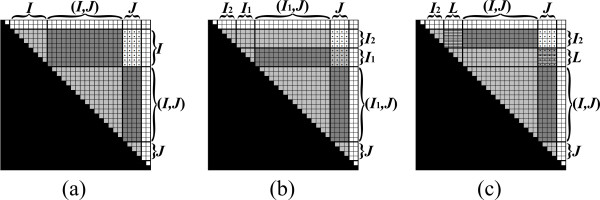
**An exemplification of the horizontal partition of *B*_*I*, *J*_**. See Figure 8 for the symmetric description of the stages.

Now, the pre-condition with respect to $B_{I,J_{2}}$ is established, and the algorithm computes $B_{I,J_{2}}$ recursively. In the case where |*I*| *>*|*J*|, *B*_*I*, *J *_is partitioned horizontally, in a symmetric manner to the vertical partition. The horizontal partition is depicted in Figure 9. The complete pseudo-code for the Inside VMT algorithm is given in Table 2.

**Table 2:** The Inside VMT algorithm

Inside-VMT (*s*)
1: Allocate a matrix *B *of size ||*s*|| × ||*s*||, and initialize all entries in *B *with *ϕ *elements.
2: Call Compute-Inside-Sub-Matrix ([0, *n*], [0, *n*]), where *n *is the length of *s*.
3: **return ***B*

Compute-Inside-Sub-Matrix (*I*, *J*)
**pre-condition: **The values in *B*_[*I*,*J*], [*I*,*J*]_, excluding the values in *B*_*I*,*J*_, are computed, and *B*_*I*,*J *_= *β*_*I*,(*I*,*J*) _⊗ *β*_(*I*,*J*),*J*_.
**post-condition: ***B*_[*I*,*J*], [*I*,*J*] _= *β*_[*I*,*J*], [*I*,*J*]_.
1: **if ***I *= [*i*, *i*] and *J *= [*j*, *j*] **then**
2: If *i *≤ *j*, compute *β*_*i*,*j *_(in *o *(||*s*||) running time) by querying computed values in *B *and the value *μ*_*i*,*j *_which is stored in *B*_*i*,*j*_. Update *B*_*i*,*j *_← *β*_*i*,*j*_.
3: **else**
4: **if **|*I*| ≤ |*J*| **then**
5: Let *J*_1 _and *J*_2 _be the two intervals such that *J*_1_*J*_2 _= *J*, and |*J*_1_| = ⌊|*J*|/2⌋.
6: Call Compute-Inside-Sub-Matrix (*I*, *J*_1_).
7: Let *L *be the interval such that (*I*, *J*)*L *= (*I*, *J*_2_).
8: Update $B_{I,J_{2}}\leftarrow B_{I,J_{2}}⊕\left(B_{I,L}⊗B_{L,J_{2}}\right)$.
9: Call Compute-Inside-Sub-Matrix (*I*, *J*_2_).
10: **else**
11: Let *I*_1 _and *I*_2 _be the two intervals such that *I*_2_*I*_1 _= *I*, and |*I*_2_| = ⌊|*I*|/2⌋.
12: Call Compute-Inside-Sub-Matrix (*I*_1_, *J*).
13: Let *L *be the interval such that *L*(*I*, *J*) = (*I*_2_, *J*).
14: Update $B_{I_{2},J}\leftarrow \left(B_{I_{2},L}⊗B_{L,J}\right)⊕B_{I_{2},J}$.
15: Call Compute-Inside-Sub-Matrix (*I*_2_, *J*).
16: **end if**
17: **end if**

#### 3.3.1 Time complexity analysis for the Inside VMT algorithm

In order to analyze the running time of the presented algorithm, we count separately the time needed for computing the base-cases of the recurrence, and the time for non-base-cases.

In the base-cases of the recurrence (lines 1-2 in Procedure Compute-Inside-Sub-Matrix, Table 2), |*I*| = |*J*| = 1, and the algorithm specifically computes a value of the form *β*_*i*,*j*_. According to the VMT requirements, each such value is computed in *o*(||*s*||) running time. Since there are Θ(||*s*||^2^) such base-cases, the overall running time for their computation is *o*(||*s*||^3^).

Next, we analyze the time needed for all other parts of the algorithm except for those dealing with the base-cases. For simplicity, assume that ||*s*|| = 2*^x ^*for some integer *x*. Then, due to the fact that at the beginning |*I*| = |*J*| = 2*^x^*, it is easy to see that the recurrence encounters pairs of intervals *I*, *J *such that either |*I*| = |*J*| or |*I*| = 2|*J*|.

Denote by *T*(*r*) and *D*(*r*) the time it takes to compute all recursive calls (except for the base-cases) initiated from a call in which |*I*| = |*J*| = *r *(exemplified in Figure 8) and |*I*| = 2|*J*| = *r *(exemplified in Figure 9), respectively.

When |*I*| = |*J*| = *r *(lines 4-9 in Procedure Compute-Inside-Sub-Matrix, Table 2), the algorithm performs two recursive calls with sub-matrices of size $r\times \frac{r}{2}$, a matrix multiplication between an $r\times \frac{r}{2}$ and an $\frac{r}{2}\times \frac{r}{2}$ matrices, and a matrix addition of two $r\times \frac{r}{2}$ matrices. Since the matrix multiplication can be implemented by performing two $\frac{r}{2}\times \frac{r}{2}$ matrix multiplications (Equation 1), *T *(*r*) is given by

$$T\left(r\right)=2D\left(r\right)+2M\left(\frac{r}{2}\right)+\Theta \left(r^{2}\right).$$

When |*I*| = 2|*J*| = *r *(lines 10-15 in Procedure Compute-Inside-Sub-Matrix, Table 2), the algorithm performs two recursive calls with sub-matrices of size $\frac{r}{2}\times \frac{r}{2}$, a matrix multiplication between two $\frac{r}{2}\times \frac{r}{2}$ matrices, and a matrix addition of two $\frac{r}{2}\times \frac{r}{2}$ matrices. Thus, *D*(*r*) is given by

$$D\left(r\right)=2T\left(\frac{r}{2}\right)+M\left(\frac{r}{2}\right)+\Theta \left(r^{2}\right).$$

Therefore, $T\left(r\right)=4T\left(\frac{r}{2}\right)+4M\left(\frac{r}{2}\right)+\Theta \left(r^{2}\right)$. By the *master theorem *[44], *T*(*r*) is given by

• *T*(*r*) = Θ(*r*^2^log^*k*+1^*r*), if *M*(*r*) = *O*(*r*^2^log^*k *^*r*) for some *k *≥ 0, and

• $T\left(r\right)=\Theta \left(M\left(\frac{r}{2}\right)\right)$, if $M\left(\frac{r}{2}\right)=\Omega \left(r^{2+\varepsilon }\right)$ for some *ε >*0, and $4M\left(\frac{r}{2}\right)\leq dM\left(r\right)$ for some constant *d <*1 and sufficiently large *r*.

The running time of all operations except for the computations of base cases is thus *T*(||*s*||). In both cases listed above, *T*(||*s*||) = *o *(||*s*||^3^), and therefore the overall running time of the algorithm is sub-cubic running time with respect to the length of the input string.

The currently best algorithms for the three standard multiplication variants described in Section 2.2 satisfy that *M*(*r*) = Ω(*r*^2+*ε*^), and imply that *T*(*r*) = Θ(*M*(*r*)). When this case holds, and the time complexity of computing the base-cases of the recurrence does not exceed *M*(||*s*||) (i.e. when the amortized running time for computing each one of the Θ(||*s*||^2^) base cases is $O\left(\frac{M\left(\left||s|\right|\right)}{||s|{|}^{2}}\right)$), we say that the problem sustains the *standard VMT settings*. The running time of the VMT algorithm over such problems is thus Θ (*M*(||*s*||)). All realistic inside VMT problems familiar to the authors sustain the standard VMT settings.

#### 3.3.2 Extension to the case where several inside properties are computed

We next describe the modification to the algorithm for the general case where the goal is to compute a series of inside property-sets *β*^1^, *β*^2^, ..., *β^K ^*(see Appendix A for an example of such a problem). The algorithm maintains a series of matrices *B*^1^, *B*^2^, ..., *B^K^*, where *B^k ^*corresponds to the inside property-set *β^k^*. Each recursive call to the algorithm with a pair of intervals *I*, *J *computes the series of sub-matrices $B_{I,J}^{1},\;B_{I,J}^{2},\;\ldots ,\;B_{I,J}^{K}$. The pre-condition at each stage is:

1. For all 1 ≤ *k *≤ *K*, all values in $B_{\left[I,J\right],\left[I,J\right]}^{k}$ are computed, excluding the values in $B_{I,J}^{k}$,

2. If a result of a vector multiplication of the form $\mu_{i,j}^{k}={⊕}_{q\in \left(i,j\right)}\left(\beta_{i,q}^{k^{\prime }}⊗\beta_{q,j}^{k^{\prime \prime }}\right)$ is required for the computation of $\beta_{i,j}^{k}$, then $B_{I,J}^{k}=\beta_{I,\left(I,J\right)}^{k^{\prime }}⊗\beta_{\left(I,J\right),J}^{k^{\prime \prime }}$.

The algorithm presented in this section extends to handling this case in a natural way, where the modification is that now the matrix multiplications may occur between sub-matrices taken from different matrices, rather than from a single matrix. The only delicate aspect here is that for the base case of the recurrence, when *I *= [*i*, *i*] and *J *= [*j*, *j*], the algorithm needs to compute the values in the corresponding entries in a sequential order $B_{i,j}^{1},\;B_{i,j}^{2},\;\ldots ,\;B_{i,j}^{K}$, since it is possible that the computation of a property $\beta_{i,j}^{k}$ requires the availability of a value of the form $\beta_{i,j}^{k^{\prime }}$ for some *k' < k*. Since *K *is a constant which is independent of the length on the input string, it is clear that the running time for this extension remains the same as for the case of a single inside value-set.

The following Theorem summarizes our main results with respect to Inside VMT problems.

**Theorem 1 ***For every Inside VMT problem there is an algorithm whose running time over an instance s is o*(||*s*||^3^)*. When the problem sustains the standard VMT settings, the running time of the algorithm is *Θ(*M*(||*s*||))*, where M*(*n*) *is the running time of the corresponding matrix multiplication algorithm over two n *× *n matrices*.

## 4 Outside VMT

In this section we discuss how to solve another class of problems, denoted *Outside VMT *problems, by modifying the algorithm presented in the previous section. Similarly to Inside VMT problems, the goal of Outside VMT problems is to compute sets of outside properties *α*^1^, *α*^2^, ..., *α^K ^*corresponding to some input string (see notations in Section 2.3).

Examples for problems which require outside properties computation and adhere to the VMT requirements are the *RNA Base Pair Binding Probabilities *problem [10] (described in Appendix A) and the *WCFG Inside-Outside *problem [43]. In both problems, the computation of outside properties requires a set of pre-computed inside properties, where these inside properties can be computed with the Inside VMT algorithm. In such cases, we call the problems *Inside-Outside VMT *problems.

The following definition describes the family of *Outside VMT *problems.

**Definition 2 ***A problem is considered an *Outside VMT *problem if it fulfills the following requirements*.

*1. Instances of the problem are strings, and the goal of the problem is to compute for every sub-instance *$\bar{s_{i,j}}$*of an input string s, a series of outside properties *$\alpha_{i,j}^{1},\;\alpha_{i,j}^{2},\;\ldots ,\;\alpha_{i,j}^{K}$.

*2. Let *$\bar{s_{i,j}}$*be a sub-instance of some input string s. Let *1 ≤ *k *≤ *K, and let *$\mu_{i,j}^{k}$*be a result of a vector multiplication of the form *$\mu_{i,j}^{k}={⊕}_{q\in \left[0,i\right)}\left(\beta_{q,i}^{k}⊗\alpha_{q,j}^{k^{\prime }}\right)$*or of the form *$\mu_{i,j}^{k}={⊕}_{q\in \left(j,n\right]}\left(\alpha_{i,q}^{k^{\prime }}⊗\beta_{j,q}^{k}\right)$*, for some *1 ≤ *k' *≤ *K and a set of pre-computed inside properties β^k^. Assume that the following values are available: *$\mu_{i,j}^{k}$*, all values *$\alpha_{i^{\prime },j^{\prime }}^{k^{\prime }}$*for *1 ≤ *k' *≤ *K and s*_*i*,*j *_*a strict substring of s*_*i'*,*j'*_*, and all values *$\alpha_{i,j}^{k^{\prime }}$*for *1 ≤ *k' < k. Then*, $\alpha_{i,j}^{k}$*can be computed in o *(||*s*||) *running time*.

*3. In the multiplication variant that is used for computing *$\mu_{i,j}^{k}$*, the *⊕ *operation is associative, and the domain of elements contains a zero element. In addition, there is a matrix multiplication algorithm for this multiplication variant, which running time M*(*n*) *over two n *× *n matrices satisfies M*(*n*) = *o*(*n*^3^).

**Figure 10:**
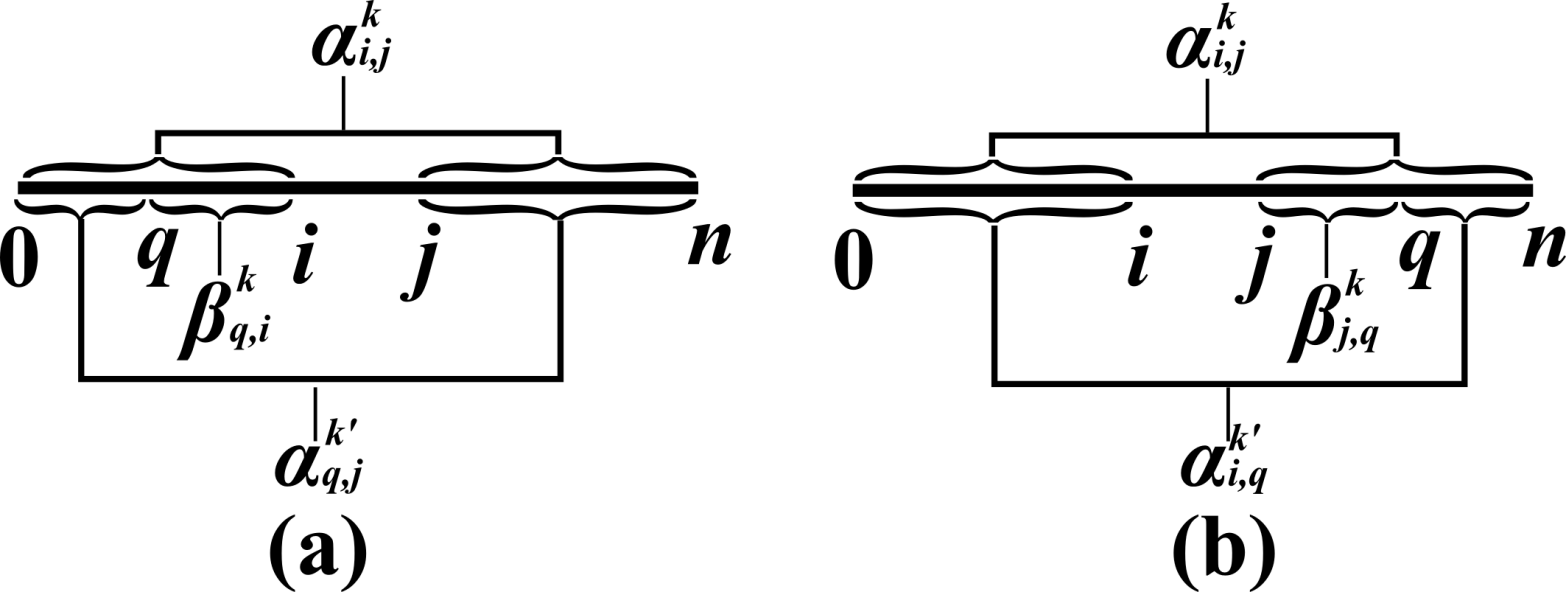
**Outside value computation**.

Here, for the case where $\mu_{i,j}^{k}={⊕}_{q\in \left[0,i\right)}\left(\beta_{q,i}^{k}⊗\alpha_{q,j}^{k^{\prime }}\right)$, the value $\mu_{i,j}^{k}$ reflects an expression which examines all possible splits of $\bar{s_{i,j}}$ into a sub-instance of the form $\bar{s_{q,j}}$, where *q < i*, and a sub-instance of the form *s*_*q*,*i *_(Figure 10a). Each split induces a term which is obtained by applying the ⊗ operation between a property $\beta_{q,i}^{k}$ of *s*_*q*,*i *_and a property $\alpha_{q,j}^{k^{\prime }}$ of $\bar{s_{q,j}}$. Then, $\mu_{i,j}^{k}$ combines the values of all such terms by applying the ⊕ operator. Symmetrically, when $\mu_{i,j}^{k}={⊕}_{q\in \left(j,n\right]}\left(\alpha_{i,q}^{k^{\prime }}⊗\beta_{j,q}^{k}\right)$, $\mu_{i,j}^{k}$ reflects a value corresponding to the examination of all splits of $\bar{s_{i,j}}$ into a sub-instance of the form $\bar{s_{i,q}}$, where *q > j*, and a sub-instance of the form *s*_*j*,*q *_(Figure 10b).

We now turn to describe a generic recursive algorithm for Outside VMT problems. For simplicity, we consider the case of a problem whose goal is to compute a single set of outside properties *α*, given a single pre-computed set of inside properties *β*. As for the Inside VMT algorithm, it is simple to extend the presented algorithm to the case where the goal is to compute a series of outside properties for every sub-instance of the input.

**Figure 11:**
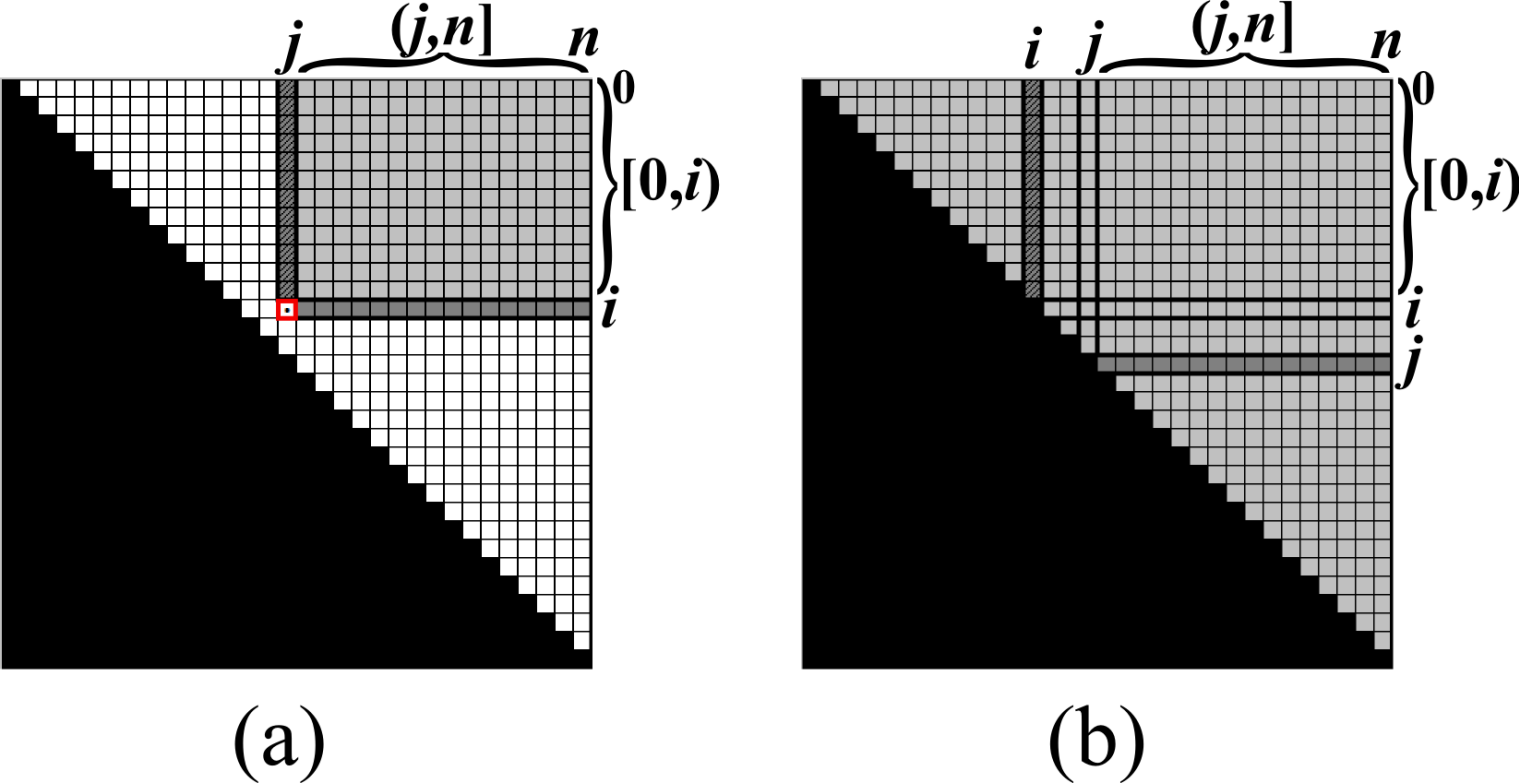
**The base case of the Outside VMT algorithm**. (a) An illustration of the matrix *A *upon computing *A*_*i*, *j*_. (b) An illustration of the pre-computed matrix *β*. All required values of the form *α*_*i'*, *j' *_for computing *α*_*i*, *j *_have already been computed in the sub-matrix *A*_[0,*i*], [*j*,*n*]_, excluding the entry *A*_*i*, *j *_itself. *μ*_*i*, *j *_is obtained either by the multiplication (*β*_[0,*i*),*i*_)*^T ^*⊗ *A*_[0,*i*), *j *_(the multiplication of the transposed striped dark gray vector in *β *with the striped dark gray vector in *A*), or by the multiplication *A*_*i*,(*j, n*] _⊗ (*β*_*j*,(*j, n*]_)*^T ^*(the multiplication of the non-striped dark gray vector in *A *with the transposed non-striped dark gray vector in *β*).

For an input string *s *of length *n*, the algorithm maintains a matrix *A *of size ||*s*|| × ||*s*||, corresponding to the required output matrix *α*. In order to compute a property *α*_*i*,*j*_, the availability of values of the form *α*_*i'*,*j'*_, such that *s*_*i*, *j *_is a strict substring of *s*_*i'*,*j'*_, is required. In terms of the matrix *A*, this means that when computing *A*_*i*, *j*_, all entries in the sub-matrix *A*_[0,*i*], [*j*,*n*]_, excluding the entry *A*_*i*, *j *_itself, need to be computed (Figure 11a).

**Figure 12:**
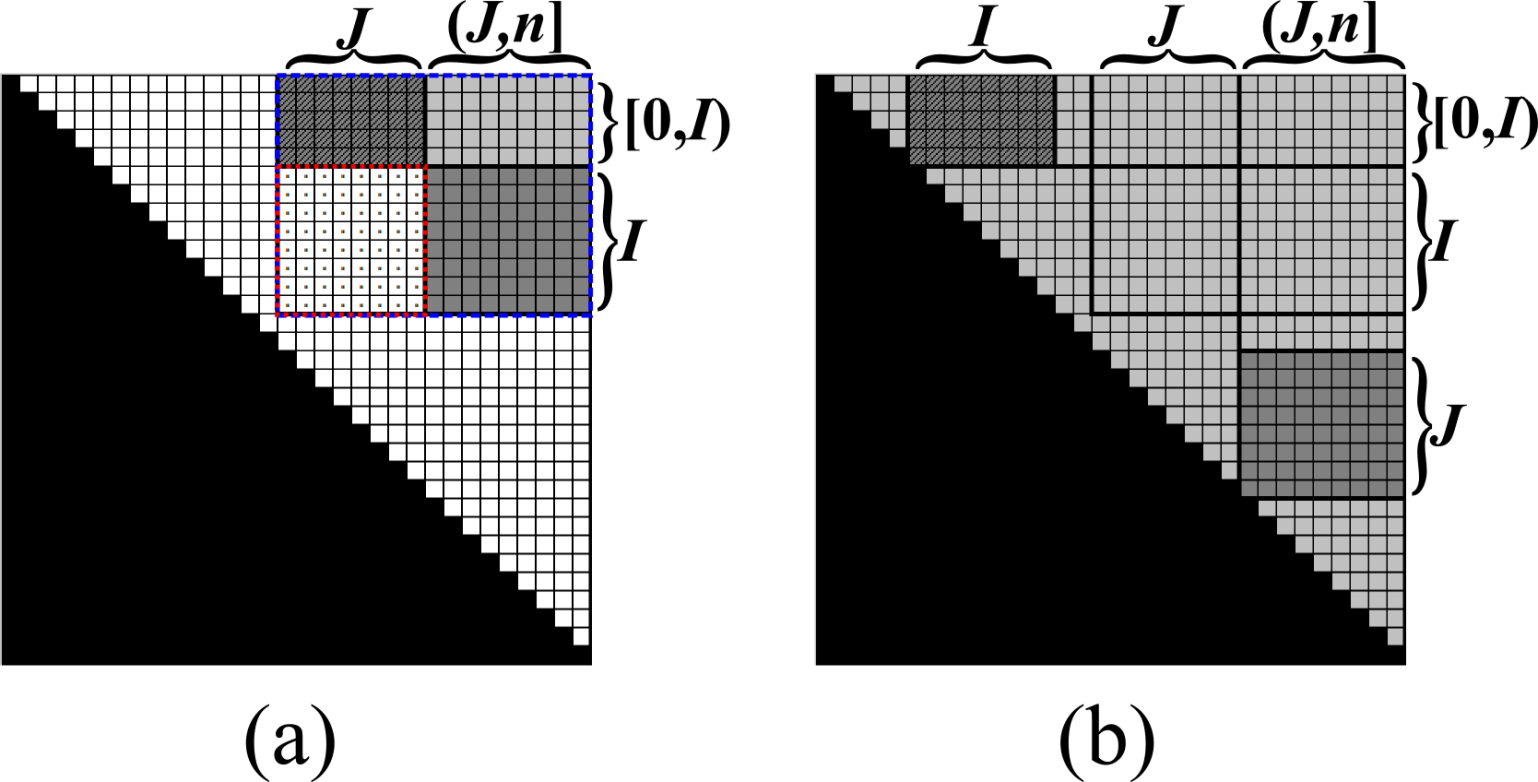
**The pre-condition of the Outside VMT algorithm**. (a) An illustration of the matrix *A *upon computing *A*_*I*, *J *_(dotted entries). (b) An illustration of the pre-computed matrix *β*. The pre-condition requires that all entries in the sub-matrix *A*_[0,*I*], [*J*,*n*]_, excluding *A*_*I*, *J*_, are computed (dark and light gray entries). In addition, *A*_*I*, *J *_is either the result of the matrix multiplication (*β*_[0,*I*),*I*_)*^T ^*⊗ *α*_[0,*I*), *J *_(the multiplication of the transposed striped dark gray matrix in *β *with the striped dark gray matrix in *A*), or the multiplication *α*_*I*,(*J, n*] _⊗ (*β*_*J*,(*J*, *n*]_)*^T ^*(the multiplication of the non-striped dark gray matrix in *A *with the transposed non-striped dark gray matrix in *β*).

At each recursive call, the algorithm computes the values in a sub-matrix of *A*. The following pre-condition is maintained when the algorithm is called over a sub-matrix *A*_*I*, *J *_(Figure 12):

1. Each entry *A*_*i*, *j *_in *A*_[0,*I*], [*J*,*n*] _contains the value *α*_*i*, *j*_, except for entries in *A*_*I*, *J*_.

2. If the computation of *α*_*i*, *j *_requires the result of a vector multiplication of the form *μ_i, j _*= ⊕_*q*∈[0,*i*) _(*β_q, i _*⊗ *α_q, j_*), then *A_I, J _*= (*β*_[0,*I*),*I*_)*^T ^*⊗ *α*_[0,*I*),*J*_. Else, if the computation of *α_i, j _*requires the result of a vector multiplication of the form *μ*_*i*, *j *_= ⊕_*q*∈(*j*,*n*] _(*α*_*i*, *q *_⊗ *β*_*j*, *q*_), then *A_I, J _*= *α*_*I*, (*j, n*] _⊗ (*β*_*j*(*J*, *n*]_)*^T^*.

**Figure 13:**
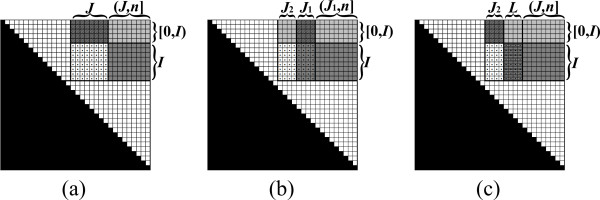
**The vertical decomposition in the Outside VMT algorithm, for the case where |*I*| ≤ |*J*|**. (a) *A*_*I*, *J *_sustains the pre-condition (see Figure 12 for notations). (b) *A*_*I*, *J *_is partitioned vertically to $A_{I,J_{1}}$ and $A_{I,J_{2}}$, where $A_{I,J_{1}}$ is computed recursively. (c) If *μ*_*i*, *j *_is of the form ⊕ _*q*∈(*j*,*n*] _(*α*_*i*, *q *_⊗ *β*_*j*, *q*_), then the pre-condition for computing $A_{I,J_{2}}$ is established, by updating $A_{I,J_{2}}$ to be $\left(A_{I,L}⊗{\left(\beta_{J_{2},L}\right)}^{T}\right)⊕A_{I,J_{2}}$ (in this example *L *= *J*_1_, since *I *ends before *J*_1 _starts. The corresponding sub-matrix of *β *is not shown). In the case where *μ*_*i*, *j *_= ⊕_*q*∈[0,*i*)_(*β*_*q*, *i *_⊗ *α*_*q*, *j*_), the pre-condition is met without any need for performing a matrix multiplication. Then, $A_{I,J_{2}}$ is computed recursively (not shown).

**Figure 14:**
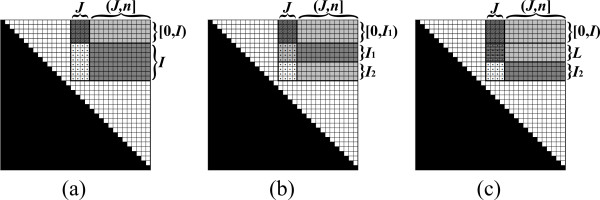
**The horizontal decomposition in the Outside VMT algorithm, for the case where |*I*| *>*|*J*|**. (a) *A*_*I*, *J *_sustains the pre-condition (see Figure 12 for notations). (b) *A*_*I*, *J *_is partitioned horizontally to $A_{I_{1},J}$ and $A_{I_{2},J}$, where $A_{I_{1},J}$ is computed recursively. (c) If *μ*_*i*, *j *_is of the form ⊕_*q*∈[0,*i*)_(*β*_*q*, *i *_⊗ *α*_*q*, *j*_), then the pre-condition for computing $A_{I_{2},J}$ is established, by updating $A_{I_{2},J}$ to be $A_{I_{2},J}⊕\left({\left(\beta_{L,I_{2}}\right)}^{T}⊗A_{L,J}\right)$ (in this example *L *= *I*_1_, since *I*_2 _ends before *J *starts. The corresponding sub-matrix of *β *is not shown). In the case where *μ*_*i*, *j *_= ⊕_*q*∈(*j*,*n*] _(*α*_*i*, *q *_⊗ *β*_*j*, *q*_), the pre-condition is met without any need for performing a matrix multiplication. Then, $A_{I_{2},J}$ is computed recursively (not shown).

The pre-condition implies that when *I *= [*i*, *i*] and *J *= [*j*, *j*], all necessary values for computing *α*_*i*, *j *_in *o*(||*s*||) running time are available, and thus *α*_*i*, *j *_can be efficiently computed and stored in *A*_*i*, *j*_. When |*I*| *>*1 or |*J*| *>*1, the algorithm follows a similar strategy as that of the Inside VMT algorithm, by partitioning the matrix into two parts, and computing each part recursively. The vertical and horizontal partitions are illustrated in Figure 13 and 14, respectively. The pseudo-code for the complete Outside VMT algorithm is given in Table 3. Similar running time analysis as that applied in the case of the Inside VMT algorithm can be shown, yielding the result stated in Theorem 2.

**Table 3:** The outside VMT algorithm

Outside-VMT (*s*, *β*)
1: Allocate a matrix *A *of size ||*s*|| × ||*s*||, and initialize all entries in *A *with *ϕ *elements.
2: Call Compute-Outside-Sub-Matrix ([0, *n*], [0, *n*]), where *n *is the length of *s*.
3: **return ***A*

Compute-Outside-Sub-Matrix (*I*, *J*)
**pre-condition: **The values in *A*_[0,*I*], [*J*,*n*]_, excluding the values in *A*_*I,J*_, are computed.
If *μ*_*i,j *_= ⊕_*q*∈[0,*i*) _(*β*_*q,i *_⊗ *α*_*q,j*_), then *A_I,J _*= (*β*_[0,*I*),*I*_)*^T ^*⊗ *α*_[0,*I*),*J*_. Else, if *μ*_*i,j *_= ⊕_*q*∈(*j*,*n*] _(*α*_*i,q *_⊗ *β*_*j,q*_), then *A_I,J _*= *α*_*I*,(*J,n*] _⊗ (*β*_*J*,(*J,n*]_)*^T^*.
**post-condition: ***A*_[0,*I*], [*J*,*n*] _= *α*_[0,*I*], [*J*,*n*]_.
1: **if ***I *= [*i*, *i*] and *J *= [*j*, *j*] **then**
2: If *i *≤ *j*, compute *α_i,j _*(in *o *(||*s*||) running time) by querying computed values in *A *and the value *μ_i,j _*which is stored in *A_i,j_*. Update *A_i,j _*← *α_i,j_*.
3: **else**
4: **if **|*I*| ≤ |*J*| **then**
5: Let *J*_1 _and *J*_2 _be the two intervals such that *J*_2_*J*_1 _= *J*, and |*J*_2_| = ⌊|*J*|/2⌋.
6: Call Compute-Outside-Sub-Matrix (*I*, *J*_1_).
7: **if ***μ_i,j _*is of the form ⊕_*q*∈(*j*,*n*] _(*α_i,q _*⊗ *β_j,q_*) **then**
8: Let *L *be the interval such that *L*(*J*, *n*] = (*J*_2_, *n*].
9: Update $A_{I,J_{2}}\leftarrow \left(A_{I,L}⊗{\left(\beta_{J_{2},L}\right)}^{T}\right)⊕A_{I,J_{2}}$.
10: **end if**
11: Call Compute-Outside-Sub-Matrix (*I*, *J*_2_).
12: **else**
13: Let *I*_1 _and *I*_2 _be the two intervals such that *I*_1_*I*_2 _= *I*, and |*I*_1_| = ⌊|*I*|/2⌋.
14: Call Compute-Outside-Sub-Matrix (*I*_1_, *J*).
15: **if ***μ_i,j _*is of the form ⊕_*q*∈[0,*i*) _(*β_q,i _*⊗ *α_q,j_*) **then**
16: Let *L *be the interval such that [0, *I*)*L *= [0, *I*_2_).
17: Update $A_{I_{2},J}\leftarrow A_{I_{2},J}⊕\left({\left(\beta_{L,I_{2}}\right)}^{T}⊗A_{L,J}\right)$.
18: **end if**
19: Call Compute-Outside-Sub-Matrix (*I*_2_, *J*).
20: **end if**
21: **end if**

**Theorem 2 ***For every Outside VMT problem there is an algorithm whose running time over an instance s is o *(||*s*||^3^)*. When the problem sustains the standard VMT settings, the running time of the algorithm is *Θ(*M*(||*s*||))*, where M*(*n*) *is the running time of the corresponding matrix multiplication algorithm over two n *× *n matrices*.

## 5 Multiple String VMT

In this section we describe another extension to the VMT framework, intended for problems for which the instance is a set of strings, rather than a single string. Examples of such problems are the *RNA Simultaneous Alignment and Folding *problem [15,37], which is described in details in Appendix B, and the *RNA-RNA Interaction *problem [9]. Additional problems which exhibit a slight divergence from the presented template, such as the *RNA-RNA Interaction Partition Function *problem [12] and the *RNA Sequence to Structured-Sequence Alignment *problem [13], can be solved in similar manners.

In order to define the Multiple String VMT variant in a general manner, we first give some related notation. An *instance *of a Multiple String VMT problem is a set of strings *S *= (*s*^0^, *s*^1^, ..., *s*^*m*-1^), where the length of a string *s^p ^*∈ *S *is denoted by *n_p_*. A *position *in *S *is a set of indices *X *= (*i*_0_, *i*_1_, ..., *i*_*m*-1_), where each index *i_p _*∈ *X *is in the range 0 ≤ *i_p _*≤ *n_p_*. The number of different positions in *S *is denoted by $\left||S||\;=\underset{0\leq p<m}{\prod }||s^{p}|\right|$.

**Figure 15:**
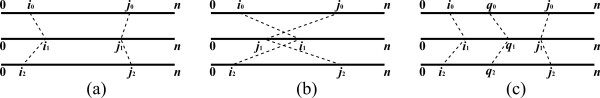
**Multiple String VMT**. (a) An example of a Multiple String VMT instance, with three strings. A sub-instance *S*_*X*, *Y *_consists of three substrings (where *X *= {*i*_0_, *i*_1_, *i*_2_} and *Y *= {*j*_0_, *j*_1_, *j*_2_}). (b) Here, since *j*_1 _*< i*_1 _we have that *X *≰ *Y*, and thus *S*_*X*, *Y *_does not correspond to a valid sub-instance. (c) A valid split of the sub-instance is obtained by splitting each one of the corresponding substrings $s_{i_{p},j_{p}}^{p}$ into a prefix $s_{i_{p},q_{p}}^{p}$ and a suffix $s_{q_{p},j_{p}}^{p}$.

Let *X *= (*i*_0_, *i*_1_, ..., *i*_*m*-1_) and *Y *= (*j*_0_, *j*_1_, ..., *j*_*m*-1_) be two positions in *S*. Say that *X *≤ *Y *if *i_p _*≤ *j_p _*for every 0 ≤ *p < m*, and say that *X < Y *if *X *≤ *Y *and *X *≠ *Y*. When *X *≤ *Y*, denote by *S*_*X*, *Y *_the *sub-instance *$S_{X,Y}=\left(s_{i_{0},j_{0}}^{0},\;s_{i_{1},j_{1}}^{1},\;\ldots ,\;s_{i_{m-1},j_{m-1}}^{m-1}\right)$ of *S *(see Figure 15a). Say that *S*_*X'*, *Y' *_is a *strict sub-instance *of *S*_*X*, *Y *_if *X *≤ *X' *≤ *Y' *≤ *Y*, and *S*_*X'*,*Y' *_≠ *S*_*X*, *Y*_.

The notation *X *≰ *Y *is used to indicate that it is not true that *X *≤ *Y*. Here, the relation '≤' is not a linear relation, thus *X *≰ *Y *does not necessarily imply that *Y < X*. In the case where *X *≰ *Y*, we say that *S*_*X*, *Y *_does not correspond to a *valid sub-instance *(Figure 15b). The notations $\bar{0}$ and *N *will be used in order to denote the first position (0, 0, ..., 0) and the last position (*n*_0_, *n*_1_, ..., *n*_*m*-1_) in *S*, respectively. The notations which were used previously for intervals, are extended as follows: [*X*, *Y*] denotes the *set *of all positions *Q *such that *X *≤ *Q *≤ *Y*, (*X*, *Y*) denotes the *set *of all positions *Q *such that *X < Q < Y*, [*X*, *Y*) denotes the *set *of all positions *Q *such that *X *≤ *Q < Y*, and (*X*, *Y*] denotes the *set *of all positions *Q *such that *X < Q *≤ *Y*. Note that while previously these notations referred to intervals with sequential order defined over their elements, now the notations correspond to sets, where we assume that the order of elements in a set is unspecified.

Inside and outside properties with respect to multiple string instances are defined in a similar way as for a single string: An inside property *β*_*X*, *Y *_is a property that depends only on the sub-instance *S*_*X*, *Y*_, where an outside property *α*_*X*, *Y *_depends on the residual sub-instance of *S*, after excluding from each string in *S *the corresponding substring in *S*_*X*, *Y*_. In what follows, we define *Multiple String Inside VMT *problems, and show how to adopt the Inside VMT algorithm for such problems. The "outside" variant can be formulated and solved in a similar manner.

**Definition 3 ***A problem is considered a *Multiple String Inside VMT *problem if it fulfills the following requirements*.

*1. Instances of the problem are sets of strings, and the goal of the problem is to compute for every sub-instance S*_*X*, *Y *_*of an input instance S, a series of inside properties *$\beta_{X,Y}^{1},\;\beta_{X,Y}^{2},\;\ldots ,\;\beta_{X,Y}^{K}$.

*2. Let S*_*X*, *Y*_* be a sub-instance of some input instance S. Let *1 ≤ *k *≤ *K, and let *$\mu_{X,Y}^{k}$*be a value of the form *$\mu_{X,Y}^{k}={⊕}_{Q\in \left(X,Y\right)}\left(\beta_{X,Q}^{k^{\prime }}⊗\beta_{Q,Y}^{k^{\prime \prime }}\right)$*, for some *1 ≤ *k'*, *k" *≤ *K. Assume that the following values are available: *$\mu_{X,Y}^{k}$, *all values *$\beta_{X^{\prime },Y^{\prime }}^{k^{\prime }}$*for *1 ≤ *k' *≤ *K and S*_*X'*, *Y'*_* a strict sub-instance of S*_*X*, *Y*_*, and all values *$\beta_{X,Y}^{k^{\prime }}$*for *1 ≤ *k' < k. Then, *$\beta_{X,Y}^{k}$*can be computed in o *(||*S*||) *running time*.

*3. In the multiplication variant that is used for computing *$\mu_{X,Y}^{k}$*, the *⊕ *operation is associative and commutative, and the domain of elements contains a zero element. In addition, there is a matrix multiplication algorithm for this multiplication variant, which running time M*(*n*) *over two n *× *n matrices satisfies M*(*n*) = *o*(*n*^3^).

Note that here there is an additional requirement with respect to the single string variant, that the ⊕ operator is *commutative*. This requirement was added, since while in the single string variant split positions in the interval (*i*, *j*) could have been examined in a sequential order, and the (single string) Inside VMT algorithm retains this order when evaluating $\mu_{i,j}^{k}$, here there is no such natural sequential order defined over the positions in the set (*X*, *Y*). The ⊕ commutativity requirement is met in all standard variants of matrix multiplication, and thus does not pose a significant restriction in practice.

Consider an instance *S *= (*s*^0^, *s*^1^, ..., *s*^*m*-1^) for a Multiple String Inside VMT problem, and the simple case where a single property set *β *needs to be computed (where *β *corresponds to all inside properties of the form *β*_*X*, *Y*_). Again, we compute the elements of *β *in a square matrix *B *of size ||*S*|| × ||*S*||, and show that values of the form *μ*_*X*, *Y *_correspond to results of vector multiplications within this matrix. For simplicity, assume that all string *s^p ^*∈ *S *are of the same length *n*, and thus ||*S*|| = (*n *+ 1)*^m ^*(this assumption may be easily relaxed).

Define a one-to-one and onto mapping *h *between positions in *S *and indices in the interval [0, ||*S*||), where for a position *X *= (*i*_0_, *i*_1_, ..., *i*_*m*-1_) in *S*,

$h\left(X\right)=\sum_{p=0}^{m-1}i_{p}\cdot {\left(n+1\right)}^{p}$. Let *h*^-1 ^denote the *inverse *mapping of *h*, i.e. *h*(*X*) = *i *⇔ *h*^-1^(*i*) = *X*. Observe that *X *≤ *Y *implies that *h*(*X*) ≤ *h*(*Y*), though the opposite is not necessary true (i.e. it is possible that *i *≤ *j *and yet *h*^-1^(*i*) ≰ *h*^-1^(*j*), as in the example in Figure 15b).

Each value of the form *β*_*X*, *Y *_will be computed and stored in a corresponding entry *B*_*i*, *j*_, where *i *= *h*(*X*) and *j *= *h*(*Y*). Entries of *B *which do not correspond to valid sub-instances of *S*, i.e. entries *B*_*i*, *j *_such that *h*^-1^(*i*) ≰ *h*^-1^(*j*), will hold the value *ϕ*. The matrix *B *is computed by applying the Inside VMT algorithm (Table 2) with a simple modification: in the base-cases of the recurrence (line 2 in Procedure Compute-Inside-Sub-Matrix, Table 2), the condition for computing the entry *B*_*i*, *j *_is that *h*^-1^(*i*) ≤ *h*^-1^(*j*) rather than *i *≤ *j*. If *h*^-1^(*i*) ≤ *h*^-1^(*j*), the property $\beta_{h^{-1}\left(i\right),h^{-1}\left(j\right)}$ is computed and stored in this entry, and otherwise the entry retains its initial value *ϕ*.

**Figure 16:**
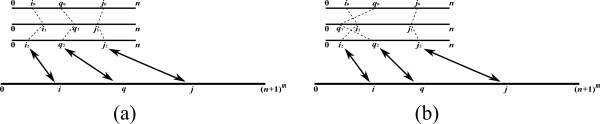
**The *h *mapping**. The notations *X*, *Y*, and *Q*, refer to the positions (*i*_1_, *i*_2_, *i*_3_), (*j*_1_, *j*_2_, *j*_3_) and (*q*_1_, *q*_2_, *q*_3_) which are illustrated in the figure, respectively. (a) A position *Q *∈ (*X*, *Y*) is mapped to an index *q *∈ (*i*, *j*), where *i *= *h*(*X*) and *j *= *h*(*Y*). (b) Some indices *q *∈ (*i*, *j*) correspond to positions *Q *= *h*^-1^(*q*) such that *Q *∉ (*X*, *Y*). This implies that either *S*_*X*, *Q *_or *S*_*Q*, *Y *_are not valid sub-instances of *S*.

In order to prove the correctness of the algorithm, we only need to show that all necessary values for computing a property *β*_*X*, *Y *_are available to the algorithm when the base-case of computing *B*_*i*, *j *_for *i *= *h*(*X*) and *j *= *h*(*Y*) is reached. Due to the nature of the mapping *h*, all properties *β*_*X'*,*Y' *_for strict sub-instances *S*_*X'*,*Y' *_of *S*_*X*, *Y *_correspond to entries *B*_*i'*, *j' *_such that *i' *= *h*(*X'*) and *j' *= *h*(*Y'*) and *i'*, *j' *∈ [*i*, *j*]. Therefore, all inside properties of strict sub-instances of *S*_*X*, *Y *_are available according to the pre-condition. In addition, at this stage *B*_*i*, *j *_contains the value *B*_*i*,(*i*,*j*) _× *B*_(*i*,*j*),*j*_. Note that for every *Q *∈ (*X*, *Y*), *q *= *h*(*Q*) ∈ (*i*, *j*) (Figure 16a), and thus *β*_*X*, *Q *_⊗ *β*_*Q*, *Y *_= *B*_*i*, *q *_⊗ *B*_*q*, *j*_. On the other hand, every *q *∈ (*i*, *j*) such that *Q *= *h*^-1^(*q*) ∉ (*X*, *Y*) sustains that either *X *≰ *Q *or *Q *≰ *Y *(Figure 16b), and therefore either *B*_*i*, *q *_= *ϕ *or *B*_*q*, *j *_= *ϕ*, and *B*_*i*, *q *_⊗ *B*_*q*, *j *_= *ϕ*. Thus, *B*_*i*, (*i*, *j*) _× *B*_(*i*,*j*),*j *_= ⊕_*Q*∈(*X*,*Y*) _(*β*_*X*, *Q *_⊗ *β*_*Q*, *Y*_) = *μ*_*X*, *Y*_, and according to the Multiple String VMT requirements, the algorithm can compute *β*_*X*, *Y *_in *o*(||*S*||) running time.

The following theorem summarizes the main result of this section.

**Theorem 3 ***For every Multiple String (Inside or Outside) VMT problem there is an algorithm whose running time over an instance S is o *(||*S*||^3^)*. When the problem sustains the standard VMT settings, the running time of the algorithm is *Θ (*M*(||*S*||))*, where M*(*n*) *is the running time of the corresponding matrix multiplication algorithm over two n *× *n matrices*.

## 6 Concluding remarks

This paper presents a simplification and a generalization of Valiant's technique, which speeds up a family of algorithms by incorporating fast matrix multiplication procedures. We suggest generic templates that identify problems for which the approach is applicable, where these templates are based on general recursive properties of the problems, rather than on their specific algorithms. Generic algorithms are described for solving all problems sustaining these templates.

The presented framework yields new worst case running time bounds for a family of important problems. The examples given here come from the fields of RNA secondary structure prediction and CFG Parsing. Recently, we have shown that this technique also applies for the *Edit Distance with Duplication and Contraction *problem [45], suggesting that it is possible that more problems from other domains can be similarly accelerated. While previous works describe other practical acceleration techniques for some of the mentioned problems [14,25,28,32-38], Valiant's technique, along with the Four Russians technique [28,30,31], are the only two techniques which currently allow to reduce the theoretical asymptotic worst case running times of the standard algorithms, without compromising the correctness of the computations.

The usage of the Four Russians technique can be viewed as a special case of using Valiant's technique. Essentially, the Four Russians technique enumerates solutions for small computations, stores these solutions in lookup tables, and then accelerates bigger computations by replacing the execution of sub-computations with queries to the lookup tables. In the original paper [29], this technique was applied to obtain an *O*(log *n*) improvement factor for Boolean Matrix Multiplication. This approach was modified in [30] in order to accelerate Min/Max-Plus Multiplication, for integer matrices in which the difference between adjacent cells are taken from a finite interval of integers. While in [30] this technique was used ad hoc for accelerating RNA folding, it is possible to decouple the description of the fast matrix multiplication technique from the RNA folding algorithm, and present the algorithm of [30] in the same framework presented here. The latter special matrix multiplication variant was further accelerated in [45] to $O\left(\frac{n^{3}}{\log^{2}n}\right)$ running time, implying that RNA folding under discrete scoring schemes (e.g. [6]) can be computed in $O\left(\frac{n^{3}}{\log^{2}n}\right)$ time, instead of in On3log3 lognlog2n time as implied by the fastest Min/Max-Plus Multiplication algorithm for general matrices [27].

Many of the current acceleration techniques for RNA and CFG related algorithms are based on sparsification, and are applicable only to optimization problems. Another important advantage of Valiant's technique is that it is the only technique which is currently known to reduce the running times of algorithms for the non-optimization problem variants, such as RNA Partition Function related problems [10,12] and the WCFG Inside-Outside algorithm [43], in which the goals are to compute the sum of scores of all solutions of the input, instead of computing the score of an optimal solution.

Our presentation of the algorithm also improves upon previous descriptions in additional aspects. In Valiant's original description there was some intersection between the inputs of recursive calls in different branches of the recurrence tree, where portions of the data were re-computed more than once. Following Rytter's description of the algorithm [42], our formulation applies the recurrence on mutually-exclusive regions of the data, in a classic divide-and-conquer fashion. The formulation is relatively explicit, avoiding reductions to problems such as transitive closure of matrix multiplication [23,26] or shortest paths on lattice graphs [42]. The requirements specified here for VMT problems are less strict than requirements presented in previous works for such problems. Using the terminology of this work, additional requirements in [23] for Inside VMT problems are that the ⊕ operation of the multiplication is commutative, and that the ⊕ operation distributes over ⊕. Our explicit formulation of the algorithm makes it easy to observe that none of the operations of the presented algorithm requires the assumption that ⊕ distributes over ⊕. In addition, it can be seen that the ⊕ operation is always applied in a left-to-right order (for non-Multiple String VMT problems), thus the computation is correct even if ⊕ is not commutative. In [42], it was required that the algebraic structure (D,⊕,⊗) would be a *semiring*, i.e. adding to the requirements of [23] an additional requirement that  D also contains an identity element 1 with respect to the ⊕ operation. Again, the algorithms presented here do not require that such a property be obeyed.

The time complexities of VMT algorithms are dictated by the time complexities of matrix multiplication algorithms. As matrix multiplication variants are essential operations in many computational problems, much work has been done to improve both the theoretical and the practical running times of these operations, including many recent achievements [24,27,40,41,46-50]. Due to its importance, it is expected that even further improvements in this domain will be developed in the future, allowing faster implementations of VMT algorithms. Theoretical sequential sub-cubic matrix multiplication algorithms (e.g. [24]) are usually considered impractical for realistic matrix sizes. However, practical, hardware-based fast computations of matrix multiplications are gaining popularity within recent years [40,41], due the highly parallelized nature of such computations and the availability of new technologies that exploit this parallelism. Such technologies were previously used for some related problems [51,52], yet there is an intrinsic advantage for its utilization via the VMT framework. While optimizing the code for each specific problem and each specific hardware requires special expertise, the VMT framework conveniently allows differing the bottleneck part of the computation to the execution of matrix multiplication subroutines, and thus off-the-shelf, hardware tailored optimized solutions can be easily integrated into all VMT problems, instead of being developed separately for each problem.

## Competing interests

The authors declare that they have no competing interests.

## Authors' contributions

SZ developed and formulated the algorithms, template definitions, and examples. MZU and DT mentored the research, contributed in writing parts of the manuscript, and were active in revising and preparing the final manuscript. All authors read and approved the final manuscript.

## Appendix

### A RNA Partition Function and Base Pair Binding Probabilities

The following example is a simplified formalization of the *RNA Partition Function and Base Pair Binding Probabilities *problem [10]. The example demonstrates the Inside-Outside VMT settings, with respect to the Dot Product variant. This problem requires the computation of several inside and outside properties for every sub-instance of the input.

As in Section 3.1, we consider the RNA folding domain. Here, instead of looking for an *optimal *folding of an input RNA string *s*, we attempt to answer the following questions:

1. For a given folding *F *of *s*, what is the probability that *s *folds spontaneously to *F*?

2. For every pair of indices *a *and *b*, what is the probability that a spontaneous folding of *s *contains the base-pair *a *• *b*?

The *Partition Function *physical model allows answering these questions. When applied to RNA folding, it is assumed that for every folding *F *of *s*, it is possible to assign a *weight *$w\left(F\right)=e^{\frac{-E\left(F\right)}{k_{B}T}}$, which is proportional to the *probability *that *s *spontaneously folds according to *F*. Here, *E*(*F*) is the energy of *F*, *k_B _*is the Boltzmann constant, and *T *is the temperature. Then, the probability that *s *folds according to *F *is given by $\frac{w\left(F\right)}{Z}$, where the normalizing factor *Z *is called the *Partition Function *and is given by

$$Z=\;\underset{\begin{array}{c} F^{\prime }\;\text{a}\;\text{folding}\\ \text{of}\;s \end{array}}{\sum }w\left(F^{\prime }\right).$$

Note that a naive computation of the RNA folding partition function would require the examination of all possible foldings of the input instance, where the number of such foldings grows exponentially with the length of the instance [53]. In [10], McCaskill showed how to efficiently compute the partition function for RNA folding in Θ(*n*^3^) running time. In addition, he presented a variant that allows the computation of base-pairing probabilities for every given pair of indices in the RNA string. We next present a simplified version of McCaskill's computation. For the sake of clarity of presentation, assume that *w*(*F*) = *e*^|*F*|^, where |*F*| is the number of base-pairs in *F*, and assume that non-complementary base-pairs are not allowed to occur. This simplification of the weight function allows focusing on the essential properties of the computation, avoiding a tedious notation.

For the "inside" phase of the computation, define two sets of inside properties *β*^1 ^and *β*^2 ^with respect to an input RNA string *s*. The property $\beta_{i,j}^{2}$ is the partition function of the substring *s*_*i*, *j*_, i.e. the sum of weights of all possible foldings of *s*_*i*, *j*_. The property $\beta_{i,j}^{1}$ is the sum of weights of all possible foldings of *s*_*i*, *j *_that contain the base-pair *i *• (*j *- 1). For *j *≤ *i *+ 1, the only possible folding of *s*_*i*, *j *_is the empty folding, and thus $\beta_{i,j}^{1}=0$ (since there are no foldings of *s*_*i*, *j *_that contain the base-pair *i *• (*j *- 1)) and $\beta_{i,j}^{2}=1$ (since the weight of the empty folding is *e*^0 ^= 1). For *j > i *+ 1, $\beta_{i,j}^{1}$ and $\beta_{i,j}^{2}$ can be recursively computed as follows.

First, consider the computation of $\beta_{i,j}^{1}$. Observe that if *s_i _*and *s*_*j *- 1 _are not complementary bases, then there is no folding of *s*_*i*, *j *_that contains *i *• (*j *- 1), and thus $\beta_{i,j}^{1}=0$. Otherwise, every folding *F *of *s*_*i*, *j *_that contains *i *• (*j *- 1) is obtained by adding the base-pair *i *• (*j *- 1) to a unique folding *F' *of *s*_*i*+1, *j*-1_, where *w*(*F*) = *e*^|*F*| ^= *e*^|*F'*|+1 ^= *e *· *e*^|*F'*|^. Therefore, $\beta_{i,j}^{1}$ is given by

$$\beta_{i,j}^{1}=\delta_{i,j-1}\beta_{i+1,j-1}^{2},$$

where *δ*_*i*, *j *- 1 _= *e *if *s_i _*and *s*_*j*-1 _are complementary bases, and otherwise *δ*_*i*,*j*-1 _= 0.

Now, consider the computation of $\beta_{i,j}^{2}$. Every folding *F *of *s*_*i*, *j *_in which *j *- 1 is paired to some index *q*, *i < q < j *- 1, is obtained by combining a folding *F' *of the prefix *s*_*i*, *q *_with a folding *F" *of the suffix *s*_*q*, *j*_, where *F" *contains the base-pair *q *• (*j *- 1). In addition *w*(*F*) = *e*^|*F*| ^= *e*^|*F'*|+|*F"*| ^= *e*^|*F'*| ^· *e*^|*F"*| ^= *w*(*F'*) · *w*(*F"*). Thus, the sum of weights of all possible different foldings of this form, denoted by $\mu_{i,j}^{2}$, is given by

$$\mu_{i,j}^{2}=\underset{q\in \left(i,j-1\right)}{\sum }\left\{\underset{\begin{array}{c} F^{\prime }\;\text{a}\;\text{folding}\\ \text{of}\;{s_{i}}_{,q} \end{array}}{\sum }\left\{\underset{\begin{array}{c} F^{\prime \prime }\;\text{a}\;\text{folding}\;\text{of}\\ s_{q,j}\;\text{which}\\ \text{contains}\;q∙\left(j-1\right) \end{array}}{\sum }\left\{w\left(F^{\prime }\right)\cdot w\left(F^{\prime \prime }\right)\right\}\right\}\right\}=\underset{q\in \left(i,j-1\right)}{\sum }\left\{\beta_{i,q}^{2}\cdot \beta_{q,j}^{1}\right\}.$$

Since $\beta_{j-1,j}^{1}=0$, $\mu_{i,j}^{2}$ can be written as $\mu_{i,j}^{2}=\underset{q\in \left(i,j\right)}{\sum }\left\{\beta_{i,q}^{2}\cdot \beta_{q,j}^{1}\right\}$.

In addition to foldings of the above form, the set of foldings of *s*_*i*, *j *_contains foldings in which *j *- 1 is unpaired, and foldings in which *j *- 1 is paired to *i*. The set of foldings of *s*_*i*, *j *_in which *j *- 1 is unpaired is exactly the set of foldings of *s*_*i*,*j*-1_, and their sum of weights is given by $\beta_{i,j-1}^{2}$. The sum of weights of all foldings in which *j *- 1 is paired to *i *is given by $\beta_{i,j}^{1}$, and thus

$$\beta_{i,j}^{2}=\mu_{i,j}^{2}+\beta_{i,j-1}^{2}+\beta_{i,j}^{1}.$$

The computation of the inside property sets *β*^1 ^and *β*^2 ^abides by the Inside VMT requirements of Definition 1 with respect to the Dot Product, and thus may be computed by the Inside VMT algorithm. The partition function of the input RNA string *s *is given by $Z=\beta_{0,n}^{2}$.

The second phase of McCaskill's algorithm computes for every pair of indices *a *and *b *in *s*, the probability that a spontaneous folding of *s *contains the base-pair *a *• *b*. According to the partition function model, this probability equals to the sum of weights of foldings of *s *which contain *a *• *b*, divided by *Z*. Therefore, there is a need to compute values that correspond to sums of weights of foldings which contain specific base-pairs. We will denote by *γ*_*i*, *j *_the sum of weights of foldings of *s *which contain the base pair *i *• (*j *- 1). The probability of a base-pair *a *• *b *is thus $\frac{\gamma_{a,b+1}}{Z}$. In order to compute values of the form *γ*_*i*, *j*_, we define the following *outside *property sets *α*^1^, *α*^2^, *α*^3 ^and *α*^4^.

A value of the form $\alpha_{i,j}^{1}$ reflects the sum of weights of all foldings of $\bar{s_{i,j}}$ that contain the base-pair (*i *- 1) • *j*. A value of the form $\alpha_{i,j}^{2}$ reflects the sum of weights of all foldings of $\bar{s_{i,j}}$ that contain a base-pair of the form (*q - *1) • *j*, for some index *q *∈ [0, *i*). A value of the form $\alpha_{i,j}^{3}$ reflects the sum of weights of all foldings of $\bar{s_{i,j}}$ that contain a base-pair of the form *j *• (*q *- 1), for some index *q *∈ (*j*, *n*], and a value of the form $\alpha_{i,j}^{4}$ reflects the partition function of $\bar{s_{i,j}}$, i.e. the sum of weights of all foldings of $\bar{s_{i,j}}$.

Every folding *F *for *s *in which the base-pair *i *• (*j *- 1) is included can be decomposed into two foldings *F' *and *F"*, where *F' *is a folding of *s*_*i*, *j *_that contains *i *• (*j *- 1), and *F" *is a folding of $\bar{s_{i,j}}$. Similarly as above, this observation implies that the sum of weights of all foldings of *s *that contain *i *• (*j *- 1) is

$$\gamma_{i,j}=\beta_{i,j}^{1}\cdot \alpha_{i,j}^{4}.$$

It is now left to show how to compute values of the form $\alpha_{i,j}^{4}$. This computation is showed next, via a mutually recursive formulation that also computes values in the sets *α*^1^, *α*^2^, and *α*^3^.

Every folding of $\bar{s_{i,j}}$ that contains (*i - *1) • *j *is obtained by adding the base-pair (*i *- 1) • *j *to a unique folding of $\bar{s_{i-1,j+1}}$. As before, this implies that

$$\alpha_{i,j}^{1}=\delta_{i-1,j}\cdot \alpha_{i-1,j+1}^{4}.$$

Every folding of $\bar{s_{i,j}}$ that contains a base-pair of the form (*q *- 1) • *j*, for some *q *∈ [0, *i*), can be decomposed into two foldings *F' *and *F"*, where *F' *is a folding of *s*_*q*, *i*_, and *F" *is a folding of $\bar{s_{q,j}}$ that contains (*q *- 1) • *j*. Again, this implies that the sum of weights of all such foldings is given by

$$\alpha_{i,j}^{2}=\underset{q\in \left[0,i\right)}{\sum }\left\{\beta_{q,i}^{2}\cdot \alpha_{q,j}^{1}\right\}.$$

Similarly, it can be shown that

$$\alpha_{i,j}^{3}=\underset{q\in \left(j,n\right]}{\sum }\left\{\alpha_{i,q}^{4}\cdot \beta_{j,q}^{1}\right\}.$$

Finally, consider the computation of $\alpha_{i,j}^{4}$. Note that the set of all foldings of $\bar{s_{i,j}}$ in which *j *is unpaired is exactly the set of all foldings of $\bar{s_{i,j+1}}$, and thus the sum of weights of all such foldings is $\alpha_{i,j+1}^{4}$. In every other folding of $\bar{s_{i,j}}$, *j *is either paired to *i *- 1, or to some index *q *- 1 such that *q *∈ [0, *i*) or *q *∈ (*j*, *n*]. Therefore,

$$\alpha_{i,j}^{4}=\alpha_{i,j+1}^{4}+\alpha_{i,j}^{1}+\alpha_{i,j}^{2}+\alpha_{i,j}^{3}.$$

It can now be verified that the computation of the property sets *α*^1^, *α*^2^, *α*^3^, and *α*^4 ^sustains all requirements from an Outside VMT problem as listed in Definition 2, therefore the Base Pair Binding Probabilities problem may be computed by the Outside-VMT algorithm.

### B RNA Simultaneous Alignment and Folding

The *RNA Simultaneous Alignment and Folding (SAF) *problem is an example of a Multiple String VMT problem, defined by Sankoff [15]. Similarly to the classical sequence alignment problem, the goal of the SAF problem is to find an alignment of several RNA strings, and in addition to find a common folding for the aligned segments of the strings. The score of a given alignment with folding takes into account both standard alignment elements such as character matchings, substitutions and indels, as well as the folding score. For clarity, our formulation assumes a simplified scoring scheme.

We use the notation of Section 5 regarding multiple string instances, positions, and sub-instances. Let *Q *= (*q*^0^, *q*^1^, ..., *q*^*m-*1^) be a position in a multiple string instance *S *= (*s*^0^, *s*^1^, ..., *s*^*m*-1^). Say that a position *Q' *= (*q'*^0^, *q'*^1^, ..., *q'*^*m*-1^) in *S *is a *local increment *of *Q *if *Q < Q'*, and for every 0 ≤ *p < m*, *q'^p ^*≤ *q^p ^*+ 1. That is, a local increment of a position increases the value of each one of the sequence indices of the position by at most 1, where at least one of the indices strictly increases. Symmetrically, say that in the above case *Q *is a *local decrement *of *Q'*. The position sets *inc*(*Q*) and *dec*(*Q*) denote the set of all local increments and the set of all local decrements of *Q*, respectively. The size of each one of these sets is *O*(2*^m^*). An *SAF instance S *is a set of RNA strings *S *= (*s*^0^, *s*^1^, ..., *s*^*m*-1^). An *alignment A *of *S *is a set of strings *A *= (*a*^0^, *a*^1^, ..., *a*^*m*-1^) over the alphabet {*A*, *C*, *G*, *U*, -} ('-' is called the *"gap" *character), satisfying that:

• All strings in *A *are of the same length.

• For every 0 ≤ *p < m*, the string which is obtained by removing from *a^p ^*all gap characters is exactly the string *s^p^*.

**Figure 17:**
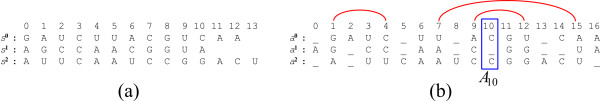
**An SAF instance, and a corresponding alignment with folding**. (a) An SAF instance *S*, composed of three RNA strings *s*^0^, *s*^1^, and *s*^2^. (b) A possible alignment with folding (*A*, *F*) of *S*, where |*A*| = 17, and *F *= {1 • 4, 7 • 15, 9 • 12}. The alignment column *A*_10 _is marked. This column corresponds to the sub-instance *S*_*Q*, *Q'*_, for *Q *= (7, 7, 8) and *Q' *= (8, 7, 9).

Denote by |*A*| the length of each one of the strings in *A*, and by $A_{r}=\left(a_{r}^{0};a_{r}^{1};\ldots ;a_{r}^{m-1}\right)$ the *r*th *column *of *A*. Observe that an alignment column *A_r _*corresponds to a sub-instance of the form *S*_*Q*, *Q'*_, where *Q' *is a local increment of *Q*. The position *Q *= (*q*^0^, *q*^1^, ..., *q*^*m*-1^) in *S *satisfies that for every 0 ≤ *p < m*, *q^p ^*is the number of non-gap characters in $a_{0,r}^{p}$. The position *Q' *= (*q'*^0^, *q'*^1^, ..., *q'*^*m*-1^) satisfies that for every 0 ≤ *p < m*, *q'^p ^*= *q^p ^*if $a_{r}^{p}$ is a gap, and otherwise *q'^p ^*= *q^p ^*+1 (see Figure 17).

A *folding F *of an alignment *A *is defined similarly to a folding of a single RNA string, except for the fact that now each pair *a *• *b *in *F *represents a column-pair in an alignment rather than a base-pair in an RNA string. Call a pair (*A*, *F*), where *A *is an alignment of an SAF instance *S *and *F *is a folding of *A*, an *alignment with folding *of *S *(Figure 17b). Define the score of an alignment with folding (*A*, *F*) to be

$$score\;\left(A,\;F\right)=\underset{0\leq r<|A|}{\sum }\rho \left(A_{r}\right)+\underset{a∙b\in F}{\sum }\tau \left(A_{a},\;A_{b}\right)$$

Here, *ρ *is a *column aligning score function*, and *τ *is a *column-pair aligning score function*. *ρ *(*A_r_*) reflects the alignment quality of the *r*th column in *A*, giving high scores for aligning nucleotides of the same type and penalizing alignment of nucleotides of different types or aligning nucleotides against gaps. *τ*(*A_a_*, *A_b_*) reflects the benefit from forming a base-pair in each one of the RNA strings in *S *between the bases corresponding to columns *A_a _*and *A_b _*of the alignment (if gaps or non-complementary bases are present in these columns, it may induce a score penalty). In addition, compensatory mutations in these columns may also increase the value of *τ*(*A_a_*, *A_b_*) (thus it may compensate for some penalties taken into account in the computation of *ρ*(*A_a_*) and *ρ*(*A_b_*)). We assume that both scoring functions *ρ *and *τ *can be computed in *O*(*m*) running time. In addition, we use as arguments for *ρ *and *τ *sub-instances of the form *S*_*Q*, *Q'*_, where *Q' *is a local increment of *Q*. In such cases, *S*_*Q*, *Q' *_corresponds to a unique alignment column, where *ρ *and *τ *are computed with respect to this column.

The goal of the SAF problem is to find the maximum score of an alignment with folding for a given SAF instance *S*. In order to compute this value, we define a set *β *of inside properties with respect to *S*, where *β*_*X*, *Y *_is the maximum score of an alignment with folding of *S*_*X*, *Y*_.

Similarly to single RNA string folding (Section 3.1), we think of two kinds of alignments with foldings for the sub-instance *S*_*X*, *Y *_: type *I *are alignments with foldings in which the first and last alignment columns are paired to each other, and type *II *are alignments with foldings in which the first and last alignment columns are not paired to each other.

**Figure 18:**
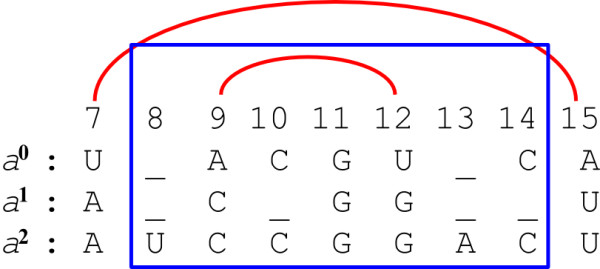
**An alignment with folding of type *I***. The alignment with folding (*A*, *F*) corresponds to the sub-instance *S*_*X*, *Y *_of the instance *S *presented in Figure 17a, where *X *= (5, 5, 5) and *Y *= (12, 10, 14). (*A*, *F*) is obtained by modifying an alignment with folding (*A'*, *F'*) for the sub-instance *S*_*X'*,*Y'*_, where *X' *= (6, 6, 6) and *Y' *= (11, 9, 13) (marked with a blue rectangle). The modification includes the addition of the two alignment columns *A*_7 _and *A*_15 _to the alignment, and the column-pair 7 • 15 to the folding.

Consider an alignment with folding (*A*, *F*) of type *I*. Let *A_a _*denote the first column in *A*, and *A_b _*the the last column in *A*. Thus, *A_a _*corresponds to some sub-instance *S*_*X*, *X'*_, where *X' *is a local increment of *X*, and similarly *A_b _*corresponds to some sub-instance *S*_*Y'*,*Y*_, where *Y' *is a local decrement of *Y*. Since *a *• *b *∈ *F*, the alignment with folding (*A*, *F*) is obtained by modifying an alignment with folding (*A'*, *F'*) for *S*_*X'*,*Y'*_, where *A *is obtained by concatenating *A_a _*and *A_b _*at the beginning and ending of *A'*, respectively, and *F *= {*a *• *b*} ∪ *F' *(Figure 18). The score of (*A*, *F*) is given by

$$score\;\left(A,\;F\right)=score\left(A^{\prime },\;F^{\prime }\right)+\rho \left(S_{X,X^{\prime }}\right)+\rho \left(S_{Y^{\prime },Y}\right)+\tau \left(S_{X,X^{\prime }},\;S_{Y^{\prime },Y}\right).$$

Therefore, it can be seen that the maximum score of an alignment with folding of type *I *is given by

$$\underset{\begin{array}{c} X^{\prime }\in inc\left(X\right)\\ Y^{\prime }\in \;dec\left(Y\right) \end{array}}{\max}\left\{\beta_{X^{\prime },Y^{\prime }}+\rho \left(S_{X,X^{\prime }}\right)+\rho \left(S_{Y^{\prime },Y}\right)+\tau \left(S_{X,X^{\prime }},\;S_{Y^{\prime },Y}\right)\right\}.$$

**Figure 19:**
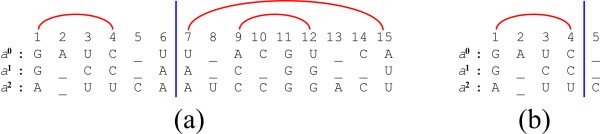
**An alignment with folding of type *II***. In such an alignment with folding the first and last alignment columns are not paired to each other, i.e. the last column is either paired to some other column (as in (a), which consists of columns 1 to 15 of the alignment with folding presented in Figure 17b), or it is unpaired (as in (b), which consists of columns 1 to 5 of the alignment with folding presented in Figure 17b). In both cases, the alignment with folding may be decomposed into two alignments with foldings (*A'*, *F'*) and (*A"*, *F"*), where (*A'*, *F'*) is an alignment with folding for *S*_*X*, *Q *_and (*A"*, *F"*) is an alignment with folding for *S*_*Q*, *Y*_, for some *Q *∈ (*X*, *Y*).

Now, consider an alignment with folding (*A*, *F*) of type *II*. As was shown for the Base-Paring Maximization problem (Section 3.1), (*A*, *F*) can be decomposed into two alignments with folding (*A'*, *F'*) and (*A*″, *F*″), where (*A'*, *F'*) is an alignment with folding of *S*_*X*, *Q1 *_and (*A*″, *F*″) is an alignment with folding of *S*_*Q*, *Y*_, for some *Q *∈ (*X*, *Y*) (Figure 19). In this case, *score*(*A*, *F*) = *score*(*A'*, *F'*) + *score*(*A*″, *F*″), and the maximum score of an alignment with folding of type *II *is

$$\underset{Q\in \left(X,Y\right)}{\max}\left\{\beta_{X,Q}+\beta_{Q,Y}\right\}.$$

Thus, *β*_*X*, *Y *_can be recursively computed according to the following formula:

$$\beta_{X,Y}=\max\left\{\begin{array}{c} \left(I\right)\;\;\underset{\begin{array}{c} X^{\prime }\in inc\left(X\right)\\ Y^{\prime }\in dec\left(Y\right) \end{array}}{\max}\;\;\left\{\beta_{X^{\prime },Y^{\prime }}\;+\rho \left(S_{X,X^{\prime }}\right)+\rho \left(S_{Y^{\prime },Y}\right)+\tau \left(S_{X,X^{\prime }},\;S_{Y^{\prime },Y}\right)\right\},\\ \left(II\right)\;\;\underset{Q\in \left(X,Y\right)}{\max}\;\;\left\{\beta_{X,Q}+\beta_{Q,Y}\right\} \end{array}\right\}.$$

In the computation of term (*I*) of the recurrence, *O*(2^2*m*^) expressions are examined, and each expression is computed in *O*(*m*) running time. Under the reasonable assumption that *m *is sufficiently small with respect to ||*S*|| (typically, *m *= 2), we can assume that *m*2^2*m *^= *o *(||*S*||), and even assume that $m2^{2m}=O\left(\frac{M\left(\left||S|\right|\right)}{||S|{|}^{2}}\right)$, where *M*(*r*) is the running time of the Max Plus multiplication of two *r *× *r *matrices.

The computation of term (*II*) is a Max Plus vector multiplication of the form *μ_X, Y _*= ⊕_*Q*∈(*X*,*Y*) _(*β_X, Q _*⊗ *β*_*Q*,*Y*_), and thus the recursive formulation abides by the standard VMT settings for the Multiple String Inside VMT requirements, as listed in Definition 3. Therefore, the SAF problem with an instance *S *can be solved in Θ (*M *(||*S*||)) = *o *(||*S*||^3^) running time. This result improves the running time of the original algorithm [15], which is Θ(||*S*||^3^).

## References

[B1] Eddy SR Noncoding RNA genes Current Opinions in Genetic Development 1999 6 6 695 699 http://view.ncbi.nlm.nih.gov/pubmed/10607607 10.1016/S0959-437X(99)00022-2 10607607

[B2] Mandal M Breaker R Gene regulation by riboswitches Cell 2004 6 451 463 10.1038/nrm1403 15173824

[B3] Griffiths-Jones S Moxon S Marshall M Khanna A Eddy S Bateman A Rfam: annotating non-coding RNAs in complete genomes Nucleic Acids Research 2005 33 Database D121 15608160 10.1093/nar/gki081 PMC540035

[B4] Consortium AFB Backofen R Bernhart SH Flamm C Fried C Fritzsch G Hackermuller J Hertel J Hofacker IL Missal K Mosig A Prohaska SJ Rose D Stadler PF Tanzer A Washietl S Will S RNAs everywhere: genome-wide annotation of structured RNAs J Exp Zoolog B Mol Dev Evol 2007 6 1 25 10.1002/jez.b.21130 17171697

[B5] Gardner P Giegerich R A comprehensive comparison of comparative RNA structure prediction approaches BMC bioinformatics 2004 6 140 10.1186/1471-2105-5-140 15458580 PMC526219

[B6] Nussinov R Jacobson AB Fast Algorithm for Predicting the Secondary Structure of Single-Stranded RNA PNAS 1980 6 11 6309 6313 10.1073/pnas.77.11.6309 6161375 PMC350273

[B7] Zuker M Stiegler P Optimal Computer Folding of Large RNA Sequences using Thermodynamics and Auxiliary Information Nucleic Acids Research 1981 6 133 148 10.1093/nar/9.1.133 6163133 PMC326673

[B8] Hofacker IL Fontana W Stadler PF Bonhoeffer SL Tacker M Schuster P Fast Folding and Comparison of RNA Secondary Structures Monatsh Chem 1994 6 167 188 10.1007/BF00818163

[B9] Alkan C Karakoç E Nadeau JH Sahinalp SC Zhang K RNA-RNA Interaction Prediction and Antisense RNA Target Search Journal of Computational Biology 2006 6 2 267 282 10.1089/cmb.2006.13.267 16597239

[B10] McCaskill JS The equilibrium partition function and base pair binding probabilities for RNA secondary structure Biopolymers 1990 6 6-7 1105 1119 10.1002/bip.360290621 1695107

[B11] Bernhart S Tafer H Mückstein U Flamm C Stadler P Hofacker I Partition function and base pairing probabilities of RNA heterodimers Algorithms for Molecular Biology 2006 6 3 10.1186/1748-7188-1-3 16722605 PMC1459172

[B12] Chitsaz H Salari R Sahinalp SC Backofen R A partition function algorithm for interacting nucleic acid strands Bioinformatics 2009 6 12 i365 373 10.1093/bioinformatics/btp212 19478011 PMC2687966

[B13] Zhang K Computing Similarity Between RNA Secondary Structures INTSYS '98: Proceedings of the IEEE International Joint Symposia on Intelligence and Systems 1998 Washington, DC, USA: IEEE Computer Society 126

[B14] Jansson J Ng S Sung W Willy H A faster and more space-efficient algorithm for inferring arc-annotations of RNA sequences through alignment Algorithmica 2006 6 2 223 245 10.1007/s00453-006-1207-0

[B15] Sankoff D Simultaneous Solution of the RNA Folding, Alignment and Protosequence Problems SIAM Journal on Applied Mathematics 1985 6 5 810 825 10.1137/0145048

[B16] Sakakibara Y Brown M Hughey R Mian I Sjolander K Underwood R Haussler D Stochastic context-free grammers for tRNA modeling Nucleic Acids Research 1994 6 23 5112 10.1093/nar/22.23.5112 7800507 PMC523785

[B17] Teitelbaum R Context-Free Error Analysis by Evaluation of Algebraic Power Series STOC ACM 1973 196 199

[B18] Dowell R Eddy S Evaluation of several lightweight stochastic context-free grammars for RNA secondary structure prediction BMC bioinformatics 2004 6 71 10.1186/1471-2105-5-71 15180907 PMC442121

[B19] Do CB Woods DA Batzoglou S CONTRAfold: RNA secondary structure prediction without physics-based models Bioinformatics 2006 6 14 e90 8 10.1093/bioinformatics/btl246 16873527

[B20] Cocke J Schwartz JT Programming Languages and Their Compilers 1970 New York: Courant Institute of Mathematical Sciences

[B21] Kasami T An efficient recognition and syntax analysis algorithm for context-free languages Tech. Rep. AFCRL-65-758, Air Force Cambridge Res. Lab., Bedford Mass 1965

[B22] Younger DH Recognition and Parsing of Context-Free Languages in Time *n*^3^ Information and Control 1967 6 2 189 208 10.1016/S0019-9958(67)80007-X

[B23] Valiant L General Context-Free Recognition in Less than Cubic Time Journal of Computer and System Sciences 1975 6 308 315 10.1016/S0022-0000(75)80046-8

[B24] Coppersmith D Winograd S Matrix Multiplication via Arithmetic Progressions J Symb Comput 1990 6 3 251 280 10.1016/S0747-7171(08)80013-2

[B25] Akutsu T Approximation and Exact Algorithms for RNA Secondary Structure Prediction and Recognition of Stochastic Context-free Languages Journal of Combinatorial Optimization 1999 6 321 336 10.1023/A:1009898029639

[B26] Benedí J Sánchez J Fast Stochastic Context-Free Parsing: A Stochastic Version of the Valiant Algorithm Lecture Notes in Computer Science 2007 6 80 88 10.1007/978-3-540-72847-4_12

[B27] Chan TM More Algorithms for All-Pairs Shortest Paths in Weighted Graphs SIAM J Comput 2010 6 5 2075 2089 10.1137/08071990X

[B28] Graham SL Harrison MA Ruzzo WL An improved context-free recognizer ACM Transactions on Programming Languages and Systems 1980 6 3 415 462 10.1145/357103.357112

[B29] Arlazarov VL Dinic EA Kronod MA Faradzev IA On Economical Construction of the Transitive Closure of an Oriented Graph Soviet Math Dokl 1970 6 1209 1210

[B30] Frid Y Gusfield D A Simple, Practical and Complete $O\left(\frac{n^{3}}{\log n}\right)$-Time Algorithm for RNA Folding Using the Four-Russians Speedup WABI 2009 6 Springer 97 107 10.1186/1748-7188-5-13 PMC2823755 20047670

[B31] Frid Y Gusfield D A Worst-Case and Practical Speedup for the RNA Co-folding Problem Using the *Four-Russians *Idea WABI 2010 1 12

[B32] Klein D Manning CD A* Parsing: Fast Exact Viterbi Parse Selection HLT-NAACL 2003 119 126

[B33] Wexler Y Zilberstein CBZ Ziv-Ukelson M A Study of Accessible Motifs and RNA Folding Complexity Journal of Computational Biology 2007 6 6 856 872 10.1089/cmb.2007.R020 17691898

[B34] Ziv-Ukelson M Gat-Viks I Wexler Y Shamir R A Faster Algorithm for Simultaneous Alignment and Folding of RNA Journal of Computational Biology 2010 6 8 1051 1065 http://www.liebertonline.com/doi/abs/10.1089/cmb.2009.0197 10.1089/cmb.2009.0197 20649420

[B35] Backofen R Tsur D Zakov S Ziv-Ukelson M Sparse RNA folding: Time and space efficient algorithms Journal of Discrete Algorithms 2010 in press http://www.sciencedirect.com/science/article/B758J-511TNF7-1/2/8d480ed24b345199f8997c1141a47d60

[B36] Salari R Mohl M Will S Sahinalp S Backofen R Time and Space Efficient RNA-RNA Interaction Prediction via Sparse Folding RECOMB 2010 6 473 490

[B37] Havgaard J Lyngso R Stormo G Gorodkin J Pairwise local structural alignment of RNA sequences with sequence similarity less than 40% Bioinformatics 2005 6 9 1815 1824 10.1093/bioinformatics/bti279 15657094

[B38] Will S Reiche K Hofacker IL Stadler PF Backofen R Inferring Non-Coding RNA Families and Classes by Means of Genome-Scale Structure-Based Clustering PLOS Computational Biology 2007 6 4 e65 10.1371/journal.pcbi.0030065 17432929 PMC1851984

[B39] Zakov S Tsur D Ziv-Ukelson M Reducing the Worst Case Running Times of a Family of RNA and CFG Problems, Using Valiant's Approach WABI 2010 65 77 10.1186/1748-7188-6-20 PMC3741081 21851589

[B40] Ryoo S Rodrigues CI Baghsorkhi SS Stone SS Kirk DB Hwu WmW Optimization principles and application performance evaluation of a multithreaded GPU using CUDA Proceedings of the 13th ACM SIGPLAN Symposium on Principles and practice of parallel programming, PPoPP '08, New York, NY, USA: ACM 2008 73 82

[B41] Volkov V Demmel JW Benchmarking GPUs to tune dense linear algebra Proceedings of the 2008 ACM/IEEE conference on Supercomputing 2008 SC '08, Piscataway, NJ, USA: IEEE Press 31:1 31:11 http://portal.acm.org/citation.cfm?id=1413370.1413402

[B42] Rytter W Context-free recognition via shortest paths computation: a version of Valiant's algorithm Theoretical Computer Science 1995 6 2 343 352 10.1016/0304-3975(94)00265-K

[B43] Baker JK Trainable grammars for speech recognition The Journal of the Acoustical Society of America 1979 6 S1 S132 S132

[B44] Bentley JL Haken D Saxe JB A General Method For Solving Divide-and-conquer Recurrences SIGACT News 1980 6 3 36 44 10.1145/1008861.1008865

[B45] Pinhas T Tsur D Zakov S Ziv-Ukelson M Giancarlo R, Manzini G Edit Distance with Duplications and Contractions Revisited CPM of Lecture Notes in Computer Science 2011 6 Springer Berlin/Heidelberg 441 454 10.1007/978-3-642-21458-5_37

[B46] Goto K Geijn R Anatomy of high-performance matrix multiplication ACM Transactions on Mathematical Software (TOMS) 2008 6 3 1 25

[B47] Robinson S Toward an optimal algorithm for matrix multiplication News Journal of the Society for Industrial and Applied Mathematics 2005 6 9

[B48] Basch J Khanna S Motwani R On diameter verification and boolean matrix multiplication Tech rep Citeseer 1995

[B49] Williams R Matrix-vector multiplication in sub-quadratic time (some preprocessing required) Proceedings of the eighteenth annual ACM-SIAM symposium on Discrete algorithms, Society for Industrial and Applied Mathematics 2007 995 1001

[B50] Bansal N Williams R Regularity Lemmas and Combinatorial Algorithms FOCS 2009 745 754

[B51] Rizk G Lavenier D Allen G, Nabrzyski J, Seidel E, van Albada G, Dongarra J, Sloot P GPU Accelerated RNA Folding Algorithm Computational Science - ICCS 2009, Volume 5544 of Lecture Notes in Computer Science Springer Berlin/Heidelberg 1004 1013

[B52] Chang D Kimmer C Ouyang M Accelerating the Nussinov RNA folding algorithm with CUDA/GPU Signal Processing and Information Technology (ISSPIT), 2010 IEEE International Symposium on IEEE 120 125

[B53] Waterman M Secondary structure of single-stranded nucleic acids Adv math suppl studies 1978 6 167 212

